# The role of consumer perspectives in estimating population need for substance use services: a scoping review

**DOI:** 10.1186/s12913-017-2153-z

**Published:** 2017-03-20

**Authors:** Elaine Hyshka, Kamagaju Karekezi, Benjamin Tan, Linda G. Slater, Jesse Jahrig, T. Cameron Wild

**Affiliations:** 1 School of Public Health, 3–300 Edmonton Clinic Health Academy, University of Alberta, 11405-87 Avenue, Edmonton, AB T6G 1C9 Canada; 2 Inner City Health and Wellness Program, B818 Women’s Centre, Royal Alexandra Hospital, 10240 Kingsway Avenue, Edmonton, AB T5H 3VR Canada; 3 John W. Scott Health Sciences Library, 2 K3.28 Walter C. Mackenzie Health Sciences Centre, University of Alberta, Edmonton, AB T6G 2R7 Canada

**Keywords:** Substance use services, Substance use disorders, System planning, Consumer perspectives, Diagnostic prevalence, Psychiatric epidemiology

## Abstract

**Background:**

A growing body of research assesses population need for substance use services. However, the extent to which survey research incorporates expert versus consumer perspectives on service need is unknown. We conducted a large, international review to (1) describe extant research on population need for substance use services, and the extent to which it incorporates expert and consumer perspectives on service need, (2) critically assess methodological and measurement approaches used to study consumer-defined need, and (3) examine the potential for existing research that prioritizes consumer perspectives to inform substance use service system planning.

**Methods:**

Systematic searches of seven databases identified 1930 peer-reviewed articles addressing population need for substance use services between January 1980 and May 2015. Empirical studies (*n* = 1887) were categorized according to source(s) of data used to derive population estimates of service need (administrative records, biological samples, qualitative data, and/or quantitative surveys). Quantitative survey studies (*n* = 1594) were categorized as to whether service need was assessed from an expert and/or consumer perspective; studies employing consumer-defined need measures (*n* = 217) received further in-depth quantitative coding to describe study designs and measurement strategies.

**Results:**

Almost all survey studies (96%; *n* = 1534) used diagnostically-oriented measures derived from an expert perspective to assess service need. Of the small number (14%, *n* = 217) of survey studies that assessed consumer’s perspectives, most (77%) measured perceived need for generic services (i.e. ‘treatment’), with fewer (42%) examining self-assessed barriers to service use, or informal help-seeking from family and friends (10%). Unstandardized measures were commonly used, and very little research was longitudinal or tested hypotheses. Only one study used a consumer-defined need measure to estimate required service system capacity.

**Conclusions:**

Rhetorical calls for including consumer perspectives in substance use service system planning are belied by the empirical literature, which is dominated by expert-driven approaches to measuring population need. Studies addressing consumer-defined need for substance use services are conceptually underdeveloped, and exhibit methodological and measurement weaknesses. Further scholarship is needed to integrate multidisciplinary perspectives in this literature, and fully realize the promise of incorporating consumer perspectives into substance use service system planning.

**Electronic supplementary material:**

The online version of this article (doi:10.1186/s12913-017-2153-z) contains supplementary material, which is available to authorized users.

## Background

Over the past four decades, significant progress has been made in the treatment of substance use disorders [1]. A large international literature shows that ‘treatment works,’ with evidence that a variety of treatment interventions and supports produce significant reductions in substance misuse and improve the health and social functioning of people seeking help for problems with alcohol and other drugs [2–5]. Despite this progress, substance use disorders continue to affect almost 150 million people worldwide [6] and many never receive care for these conditions. Misuse of alcohol and other drugs is a growing cause of noncommunicable disease burden, accounting for 2.9 million deaths and over 120 million disability-adjusted life-years (DALYs) in 2010 alone [7, 8]. Alleviating suffering by closing this treatment gap has been declared a global mental health priority [9].

In the United States and elsewhere, concerted efforts to scale up population access to substance use services and supports began in the 1980s [10, 11]. These efforts were accompanied by studies attempting to estimate population need for care in relation to existing service coverage. This reflected a shift away from research characterizing the demographic and clinical correlates of treatment-seekers, toward community-based studies of need for services in both general and special populations, regardless of whether general health care or specialty addiction treatment was actually sought or received [12].

### Conceptualizing population need for substance use services

Initial approaches used indirect estimation techniques designed to quantify need for services in relation to the prevalence of substance use and misuse in the general population, using a variety of statistical models applied to administrative datasets (e.g., retail alcohol sales, mortality data, HIV testing databases) [13–16]. However, administrative datasets have limited capacity to inform service system planning because they typically do not differentiate between subpopulations that might benefit from different types of services in relation to problem severity, e.g., substance misusers versus those meeting diagnostic criteria for substance use disorders; or people who do and do not exhibit comorbid substance use and mental disorders. These limitations hinder efforts to estimate how many people in the general population could benefit from accessing the variety of treatment interventions and supports for substance misuse typically offered by general healthcare and speciality service systems (e.g., brief interventions, outpatient care, speciality residential treatment) [14].

More recently, research turned to direct estimation methods using community-based population surveys. These approaches attempt to quantify population need for services in relation to prevalence of substance use disorders, as assessed by structured diagnostic instruments (e.g., the Composite International Diagnostic Interview [CIDI]) [12, 17]. Two American studies, the Epidemiological Catchment Area Program (ECA; 1980–1985) [18] and the first iteration of the National Co-Morbidity Study (NCS; 1990–1992) [19] were early examples of this approach. These seminal studies clearly documented that most people meeting diagnostic criteria for substance use disorders do not access health services. Past year general health care and specialty treatment rates in the ECA were 23.6% for respondents meeting diagnostic criteria for any substance use disorder [18], and varied between 11.6% (alcohol abuse) to 46.8% (drug dependence) in the NCS [20]. Other countries have since implemented large population-based mental health surveys with similar results [17, 21].

### *Expert* versus *consumer perspectives on population need for services*

These consistent findings of large treatment gaps for substance use and other mental health problems led to questions about the validity of diagnostic systems used in population-based studies of service need. Some speculated that disorder prevalence estimates were inflated, and not a true reflection of actual need for care in the population [11, 12]. Several changes were subsequently made to the fourth edition of the Diagnostic and Statistical Manual (DSM-IV) and the CIDI in order to provide information to complement diagnoses, including clinical significance, impairment, and disability [22–24]. Although these changes yielded more conservative prevalence estimates [24], they continued to prioritize an expert perspective on population need for services. This priority has been called into question, however, in light of growing evidence that diagnostically-assessed need for care is only modestly predictive of actual service use [11, 24–27]. For example, Demyttenaere and colleagues [25] report that across seven high-income countries, 36 to 50% of respondents meeting diagnostic criteria for severe substance use and mental disorders had no past year service use; and that most individuals receiving care were ‘subthreshold’ cases that exhibited low-severity problems, or failed to meet diagnostic criteria [11, 27].

From a broader health system perspective, deinstitutionalization of mental health and substance use services, cost-cutting and increasing reliance on community-based mental health supports, and the growth of countercultural anti-psychiatry and mental health survivor movements in the later half of the 20th century precipitated a shift in thinking regarding the role of patients in the mental health system [28]. Ex-patient voices gained prominence in the wake of failed system reforms, and began challenging medical authority including involuntary confinement and aggressive treatment approaches. New service delivery models emerged that increasingly conceptualized patients as active participants in mental health care, with insight on their own conditions, and the ability to make treatment decisions based on their own sense of well-being [28]. These models imply that assessing population need for care for substance misuse is a socially negotiated process that is at least partially determined by consumers’ perceptions of need for care [29–31]. The importance of consumer-defined need for services echoes Andersen’s [32] influential model of healthcare utilization, wherein predisposing and enabling factors affect individual perceptions of healthcare needs, and these perceptions are theorized to be a proximal determinant of service use. From this perspective, assessing consumers’ subjective views on service need is at least as important as determining diagnostic prevalence rates for researchers attempting to quantify population need for services with an eye toward service system planning [29, 30]. Two related concepts emphasizing a consumer perspective: self-assessed barriers to care [33], and help - seeking from friends or family members (potentially in lieu of formal service use) [34], have also been examined in this regard.

Emerging research incorporating consumer perspectives on service need demonstrates that only about 10–30% of individuals who meet objective diagnostic criteria for substance use disorders actually perceive a need for care [35–38], and that perceiving a need for care is strongly associated with service use [39]. Individuals who do perceive a need for services, but do not have this care need met, report high levels of disability and distress [40], which tend to improve once care needs are met [36]. However, 5–21% of individuals who access services, nevertheless still report service needs have not been met [35, 36, 40–42]. Research on consumer-defined need for services also demonstrates that motivational or attitudinal barriers--such as a desire to self-manage one’s own symptoms--appear to be more common than cost or other structural barriers, as reasons for having unmet care needs [33, 43, 44]. Collectively, the above findings suggest that expert-defined measures of population need (i.e. prevalence of alcohol and other drug misuse, prevalence of substance use disorders, and/or service utilization rates) are necessary, but may be insufficient for generating robust estimates of population need for services.

### Research aims

Direct estimation using surveys has become a recommended approach for making inferences about population need for substance use services [27]. Several studies estimate population need for substance use services using an expert perspective, and use the results to estimate required service system capacity [45–47]. However, many have called for complementing diagnostically-oriented approaches to direct estimation with consumer-defined need measures [27, 45], since they have the potential to provide a more complete assessment of population need for services [48–50].

Unfortunately, to our knowledge, no previous reviews have systematically mapped the large interdisciplinary literature on approaches to assessing population need for substance use services, or assessed the extent to which consumer perspectives have been incorporated. Moreover, no research has taken stock of the variety of measurement approaches used to assess consumer-defined need, nor the utility of these studies for informing service system planning. To address this gap, we conducted a systematic scoping review to critically analyze the literature describing population need for substance use services. Scoping reviews “aim to map rapidly the key concepts underpinning a research area, and the main sources and types of evidence available” ([51], p. 194). They are particularly appropriate when, as here, an area of literature is interdisciplinary, conceptually complex, and has not been systematically reviewed before. Our specific aims were to (1) describe extant research on population need for substance use services, and the extent to which it incorporates expert and consumer perspectives on service need, (2) critically assess the methodological and measurement approaches used to study consumer-defined need for substance use services, and (3) examine the potential for existing research that prioritizes consumer perspectives to inform substance use service system planning.

## Methods

Our study protocol was adapted from Arksey and O’Malley’s [52] scoping review framework. Although our review was not intended to assess study quality or synthesize research evidence, the PRISMA reporting guidelines for systematic reviews [53] informed the reporting of our results.

### Search strategy

LS (a professional health sciences research librarian) led the development of our systematic search strategy and executed the final search. We conducted multiple test searches using an *a priori* list of keywords and subject headings to develop and refine database-specific controlled vocabularies (Additional file 1) designed to identify relevant studies using a broadly inclusive approach based on two central concepts: ‘substance use services’ and ‘population need assessment.’ We searched seven databases including Medline®, Embase®, PsycInfo, EBSCO CINAHL Plus®, Scopus®, Web of Science® Core Collection, and Evidence-based Medicine Reviews® to maximize the breadth of our results. The scope was limited to English language, peer-reviewed journal articles and book chapters [not editorials/commentaries, conference abstracts, letters, or dissertations] published between 1980 and May 28, 2015 and available via the University of Alberta holdings. Our search identified 23,005 records. A total of 8736 duplicates were removed, leaving a sample of 14,269 unique records.

### Screening

**Fig. 1:**
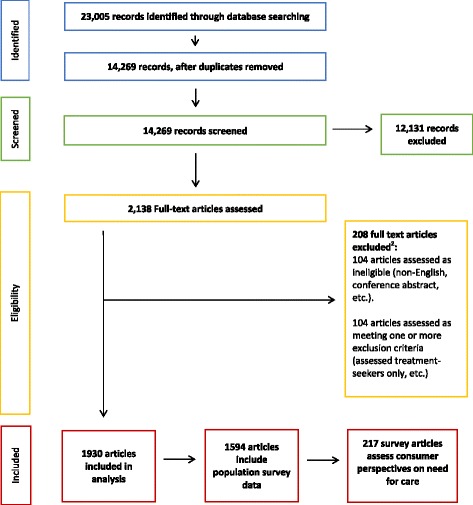
Systematic search strategy flow diagram. Adapted from PRISMA 2009 Flow Chart (The PRISMA Group, 2009). 208 full text articles excluded for the following reasons: assessed treatment-seekers only (*n* = 56); focus on general health status or other health problem (e.g., need for HIV care) (*n* = 28); mental health only, excludes substance use (*n* = 3); non-empirical, not relevant to assessment of need (*n* = 2); did not assess population need directly (e.g., survey of administrators' opinions on need) (*n* = 6); intervention study (*n* = 9); non-English (*n* = 14); not a journal article (e.g., commentary) (*n* = 17); full text could not be retrieved (*n* = 73)

All titles and abstracts were screened for relevance. Comprehensive inclusion and exclusion criteria are outlined in Table 1. EH, KK, and BT screened the records. To ensure consistency in the screening process, all raters assessed and monitored inter-rater agreement on triaging decisions using Light’s Kappa coefficient [54, 55]. Instances of disagreement were discussed with JJ and TCW and the inclusion and exclusion criteria were refined and clarified, as necessary. Raters co-screened 10 consecutive batches of 50 records until acceptable inter-rater agreement on article screening was reached (Light’s Kappa = 0.8). Screening then proceeded independently among the raters, although eight additional random batches of 50 were co-screened periodically to verify that acceptable agreement was maintained throughout the screening stage (Light’s kappa = 0.8 or greater). The title and abstract screening stage identified 2138 potentially relevant records. The full text of each record was then reviewed and relevant data extracted based on a standard coding framework. During this process, an additional 208 articles were determined to be ineligible or met exclusion criteria. Figure 1 reports our systematic search strategy flow diagram in accordance with PRISMA guidelines [53]. The remaining 1930 articles are included within the scope of this review.

**Table 1:** Scoping review inclusion and exclusion criteria

Articles were included if they:
1. Measured population need for substance use services using expert-defined (use prevalence, diagnostic prevalence, service utilization) and/or consumer-defined measures (perceived need, barriers to care, or help-seeking from family and friends); and
2. Recruited samples from community settings; and/or
3. Outlined one or more methods for assessing population need for substance use services or estimating required substance use service system capacity; and/or
4. Reviewed literature on methodological or conceptual issues in assessing population need for substance use services.
Articles were excluded if they:
1. Described need for substance use services exclusively amongst treatment-seeking or clinical samples; or
2. Described need for services not intended to mitigate substance use disorders/problematic use directly (e.g. Hepatitis C treatment, HIV prevention programs, dental care); or
3. Described service needs among populations experiencing mental disorders only (i.e. excluded substance use disorders); or
4. Were effectiveness or cost-effectiveness studies; or
5. Narratively summarized empirical findings of previous research on population need for substance use services.

### Data extraction

We developed a coding framework for included articles based on knowledge of the literature and preliminary analysis of included articles (see Additional file 2). EH coded all articles for: single or cross-national study; country; country type (high, middle or low income); global region; and whether or not empirical data were reported. All empirical articles (*n* = 1887) were then coded according to the type (s) of data used to derive population estimates of service need (administrative, biological, qualitative, or quantitative survey). All empirical studies using quantitative survey data (*n* = 1594) were classified according to whether they used one or more expert-defined measures (i.e., substance use prevalence, substance use disorder prevalence, service utilization rates) and/or one or more consumer-defined measures (i.e., perceived need for services, self-assessed barriers to service use, and/or self-reported help-seeking from family or friends).

In-depth coding was conducted on all studies containing one or more measures of consumer-defined need for services (*n* = 217; see Additional file 3, which contains a complete list of articles presenting one or more measures of consumer-defined need). These articles were coded for: study design; target population; participant characteristics; measurement (single, multi-item and standardized measures) and analytical approaches (descriptive vs. hypothesis-driven). We also coded whether or not each study estimated required service system capacity using one or more measures of consumer-perceived need for services. All articles containing consumer-defined measures were double coded by KK and BT. Disagreements were discussed with EH and corrected as appropriate.

## Results

**Fig. 2:**
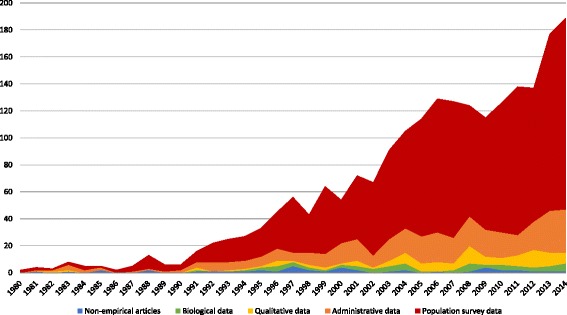
Frequency of studies addressing population need for substance use services by data type, and year (*n* = 1930). Thirty-nine studies published between January 1 and May 28 2015 were excluded from this figure due to incomplete data for 2015

Our scoping review identified 1930 articles. Figure 2 describes these articles by data source and publication year.

Overall, 1887 articles reported empirical data: 83% (*n* = 1594) estimated population need for services using quantitative survey data, 20% (*n* = 380) used administrative data, 6% (*n* = 116) used qualitative data, and 3% (*n* = 62) used biological samples (e.g., urinalysis). These percentages do not sum to one hundred, as a subset of these articles (13%; *n* = 251) used more than one type of data.

**Fig. 3:**
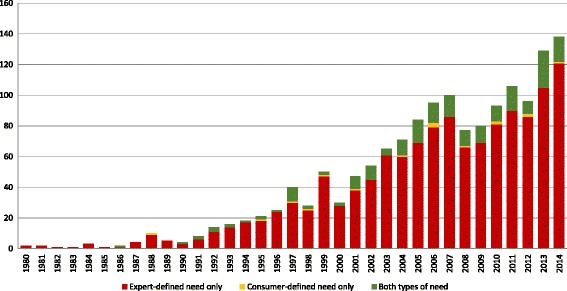
Frequency of survey research studies containing one or more measures of expert-defined need, consumer-defined need, or both expert and consumer-defined need, by publication year (*n* = 1594). Expert-defined need: reporting one or more of: prevalence of substance use, prevalence of substance use disorders (using some expert diagnostic criteria), and/or substance use service utilization rates. Note that ‘user-only’ studies (i.e., studies where eligibility criteria included substance use) were only coded as reporting a substance use prevalence estimate when survey data were combined with administrative data to estimate population prevalence of use. Consumer-defined need: reporting one or more of: perceived need for substance use services, self-assessed barriers to accessing substance use services, and/or informal help-seeking from family or friends. 36 studies published between January 1 and May 28 2015 were excluded from this figure due to incomplete data for 2015

Almost all population survey studies (95%; *n* = 1512) reported data collected from within one country only. Amongst these studies, 88% (*n* = 1335) were conducted in high-income countries, with over half (56%; *n* = 853) in the United States alone. The vast majority (84%; *n* = 1334) of population survey studies were published between 2000 and 2015, with 38% (*n* = 611) published since the start of 2010. Figure 3 describes the extent to which survey studies (*n* = 1594) used expert and/or consumer-defined approaches to estimate population need for substance use services, over time. In total, 96% (*n* = 1534) of survey research articles reported data derived from one or more expert-defined measures, and 84% (*n* = 1333) reported expert-defined estimates exclusively. In contrast, only 14% (*n* = 217) of all survey research articles reported data derived from one or more consumer-defined measures of need for service. The majority (93%; *n* = 201) of the articles reporting consumer-defined need estimates also included expert-defined need estimates. Only 16 articles (7%) reported consumer-defined estimates exclusively. A small number (3%; *n* = 44) of population survey studies met study inclusion criteria, but did not use either expert or consumer-defined need measures. Examples of these articles include studies that described demographic (or other characteristics) of community-based substance users [56], but not service need.

**Fig. 4:**
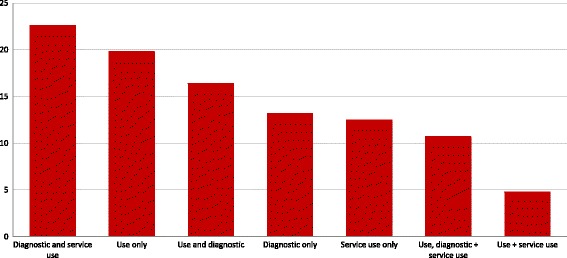
Percent of expert-defined service need studies using specific combinations of measures (*n* = 1534). Use refers to substance use prevalence, defined as an estimate of the population prevalence of one or more types of substance use. Note that ‘user-only’ studies (i.e., studies where eligibility criteria included substance use) were only coded as reporting a substance use prevalence estimate when survey data were combined with administrative data to estimate population prevalence of use. Diagnostic refers to diagnostic prevalence defined as meeting criteria for one or more substance use disorders or problematic patterns of substance use. Service use is defined as reporting use of one or more substance use services (general health, social, or specialty substance use and mental health services

Amongst expert-defined need studies, 63% (*n* = 965) reported diagnostic prevalence, 52% (*n* = 794) reported substance use prevalence, and 51% (*n* = 776) reported rates of service use. Figure 4 illustrates how expert-defined need has been conceptualized in the literature on population need for substance use services. Almost one quarter of studies (23%; *n* = 346) report both diagnostic prevalence and service use estimates. However, studies reporting only estimates of substance use prevalence (20%; *n* = 304) are also common. Only 10.7% (*n* = 165) of studies report data derived from all three expert-defined need measures.

### Consumer-defined approaches to measuring population need for substance use services

#### Target populations

In-depth coding of consumer-defined need studies indicated that 36% (*n* = 77) recruited ‘user-only’ samples, where participants were required to report substance use to be eligible for the study. Most of these studies recruited unspecified drug users (31%; *n* = 24), injection drug users (25%; *n* = 19), or stimulant users (22%; *n* = 17). Studies conducted with representative samples of general adult populations also comprised a significant proportion (35%; *n* = 76) of research on consumer-defined need for substance use services. Of these, 83% (*n* = 63) reported findings from the US, 8% (*n* = 6) from Canada, 3% (*n* = 2) from Australia, with the remaining proportion comprised of individual studies from New Zealand, the United Kingdom, Mexico, China, and South Korea. Additionally, 30% (*n* = 64) of consumer-defined need studies were conducted amongst members of special populations. The most common special populations studied were adults and youth involved in criminal justice systems (34%; *n* = 24) and people experiencing homelessness (25%; *n* = 16). Sample sizes ranged between 18 and 336,003. In terms of sample demographics, most (86%; *n* = 185) consumer-defined need studies included both male and female participants. The mean age of participants ranged from 13 to 75 (amongst the 108 studies where age was reported). Finally, with regards to study design, the majority of consumer-defined need studies adopted a cross-sectional (86%; *n* = 187) rather than longitudinal design, and produced descriptive analyses (79%; *n* = 172), rather than testing specific hypotheses.

#### Measurement approaches

**Fig. 5:**
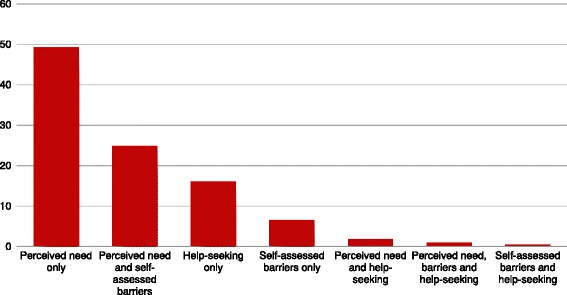
Percent of consumer-defined service need studies using specific combinations of consumer-defined need measures (*n* = 217). Perceived need defined as an individuals’ judgments about whether they require substance use services. Self-assessed barriers defined as an individual’s judgment regarding factors that impede substance use service utilization. Help-seeking defined as self-reporting seeking help from family or friends for substance use problems

Most studies (77%; *n* = 167) assessing consumer perspectives reported perceived need estimates, with far fewer measuring self-assessed barriers to service use (42%; *n* = 92), or help - seeking from family or friends (10%; *n* = 21). Overall, 72% (*n* = 156) of these studies included only one consumer-defined need measure and only two studies (1%) examined all three measures together. Figure 5 illustrates the variety of ways consumer-defined need for care was assessed.

A variety of single, multiple item, and standardized measures were used to estimate consumer-defined need for services. The majority of studies measuring perceived need (68%; *n* = 113), and studies measuring help - seeking (71%; *n* = 15) assessed these constructs using single item measures. Just over half (51%; *n* = 92) of studies measuring self-assessed barriers to service use adopted multiple-item measures (measurement details were not specified in 19% (*n* = 14) of these studies). Table 2 illustrates the diversity of single and multiple item measures identified. Consumer-defined need measures tended to define substance use services using a variety of different terms and phrases. For example, amongst studies assessing perceived need for substance use services, 84% (*n* = 140) of measures referred to generic ‘treatment’ only, whereas the remaining 26% (*n* = 27) of studies asked about need for one or more specific service types (e.g. counselling, pharmacotherapy, syringe exchange). Very few survey research articles in our review employed standardized instruments to measure consumer-defined service need. For example, a standardized instrument was used in only 7% (*n* = 12) of articles measuring perceived need. Table 3 provides a complete list of all standardized consumer-defined need measures uncovered in our review.

**Table 2:** Examples of measures used to assess consumer-defined need for substance use services (*n* = 217)

Single item measures (*n* = 113)	
Perceived need	“*Do you feel you could use treatment for drug or alcohol use*?” [67]
	“*During the past 12 months*, *was there ever a time when you felt that you needed help for your emotions*, *mental health*, *or use of alcohol or drugs*, *but you didn*’*t receive it*?” [40]
	Participants were read the following statement: “*I now need to get into a drug abuse treatment program*.” They were asked to answer along on a 5 point scale ranging from “strongly disagree” to “strongly agree.” [68]
Self-assessed barriers to service use	“*Have you ever thought you should seek help for drinking*, *but you did not go*?” Those answering yes were queried about the reasons for not seeking treatment. Answers were grouped into financial, structural, attitudinal and other categories [69].
	For all persons who reported having difficulty accessing needles, the following question was then asked: “*If yes or sometimes*, *why do you find it hard to get new* [*unused*] *rigs*?” The interviewer did not read out a list of possible explanations, but had a list of nine possible responses as well as space to note answers that did not fit with one of the nine categories [70].
Help-seeking from family and/or friends	“*On the last occasion*, *how did you try to change your drug use*?” (response categories: by myself; with family/friends; home detoxification; residential detoxification; methadone; doctor; counsellor; Alcoholics Anonymous) [71]
	Participants were asked whether they had used a range of smoking-cessation supports and aids in the last year (family and friends were one source queried) [72]
Multiple item measures (*n* = 52)	
Perceived need	Participants were asked three items with five-point response scales ranging from strongly disagree to strongly agree: (I) “*In terms of the things I need right now*, *getting into drug abuse treatment is at the top of the list*”; (*2*) “*I now need to get into drug abuse treatment for drug addiction*”; *and* (*3*) “*Because of my drug use*, *I now need drug abuse treatment*.” The sum of the scores on these items comprised a composite measure of the perceived need for treatment. Scores could range from 3 to 15 [73].
	“*Did you think you needed help for alcohol or drug problems*?” Those with a perceived need were asked, “*Were there any times during the past 12 months when you got less treatment for emotional*, *mental health*, *alcohol*, *or drug problems than you needed*, *or had difficulties or delays in getting care*?” [74]
Self-assessed barriers to service use	Administered checklist of 36 commonly cited reasons for not seeking treatment for alcohol and drug dependence. Items pertain to areas like feelings, coping with stress (family, financial and personal), perceived useful effects of drugs, cost of treatment, perceived effectiveness, treatment related fears and social reasons. The answers were recorded as Yes/No [75].
	“*Veterans may face obstacles getting or using mental health services for a number of reasons. Please rate how much you agree or disagree with each statement as it applies to you*.” 17 statements related to treatment effectiveness (e.g., “*I don’t think treatment will help me*”), stigma (e.g.,﻿ “*I would be seen as weak by others*”), and external barriers (“*It’s hard getting time off work for treatment*”) were listed. Responses ranked on a 4-point scale: 1, strongly disagree; 2, somewhat disagree; 3, somewhat agree; and 4, strongly agree [76].
Help-seeking from family and/or friends	Participants were asked to rank the perceived helpfulness of 34 interventions and whether they were used in the previous two years. “Close friend” and “close family” were two interventions listed [34].
	Participants were asked whether they had sought help from family and/or friends to reduce/cease methamphetamine use in the past 30 days. A second measure asked about help from family and/or friends in the past 12 months [77].

Perceived need defined as an individual’s judgments about whether they require substance use services. Self-assessed barriers defined as an individual’s judgment regarding factors that impede substance use service utilization. Help-seeking defined as self-reporting seeking help from family or friends for substance use problems. Note that two studies which reported perceived need estimates did not specify whether a single or multi-item measure was used

**Table 3:** Standardized measures used to assess consumer-defined need for substance use services (*n* = 217)

*Variable*	*Instrument*	*Cited in*
Perceived need	*Texas Christian University*’*s Self*-*Rating Psychosocial Functioning and Motivational Scales* [78]	[79]
	*Stages of Change Readiness and Treatment Eagerness Scale*- *Version 8 Combined* [80]	[80]
	*Revised Risk Behaviour Assessment* [81]	[82]
	*Perceived Need for Care Questionnaire* [30]	[36, 83, 84]
	*National Technical Centre* (*NTC*) *Telephone Substance Dependence Needs Assessment Questionnaire* [85]	[86]
	*Self*-*help and Treatment Services Utilization Survey* [87]	[88]
	*Camberwell Assessment of Need* - *Forensic Short Version* [89]	[90, 91]
	*University of Miami Health Services Research Instrument* [92]	[92]
Self-assessed barriers to service use	*Affordability barriers scale* [93]	[94–96]
	*Allen Barriers to Treatment Instrument* [97]	[98]
	*Barriers Questionnaire* [99]	[100]
	*Barriers to Treatment Instrument* [101]	[102]
	*Barriers to Treatment Inventory* [103]	[104]
	*University of Miami Health Services Research Instrument* [92]	[92]
Help-seeking from family and/or friends	*Mental and Physical Health Questionnaire* [105]	[105]
	*Michigan Alcoholism Screening Test* [106]	[107]

In some cases, one or more measures, but not all measures included in the standardized instrument where used to measure consumer-defined need. One article measuring perceived need did not specify the name of the standardized instrument used

As outlined at the start of this review, measuring consumer-defined need for services is an important endeavor for improving understanding of the substance use disorder treatment gap, and estimates of perceived need, in particular, have the potential to enhance substance use service system planning. We examined the extent to which consumer-defined need studies have incorporated data on perceived need into estimates of required system capacity to meet population need for substance use services. We identified only one study (*n* = 1; 0.1%) where perceived need estimates were used to calculate required service system capacity. The study was published in 1991 [57] and used telephone survey data to estimate the number of residential substance use treatment spaces needed to meet existing levels of perceived need in Rhode Island.

Given the lack of required system capacity estimates in the perceived need literature, we also considered the extent to which data reported in these articles could *hypothetically* be used to inform system planning. To do this, we calculated how many articles contained, at minimum, an estimate of substance use disorder prevalence, service utilization, and perceived need for a given sample or population. We selected these variables because they are the most basic measures required to incorporate perceived need into estimates of required service system capacity [49]. In total, 63% (*n* = 106) of all perceived need articles contained all three measures, and could potentially be used to inform substance use service planning. Half of these articles (*n* = 54) were derived from general population surveys.

## Discussion

This scoping review assessed the extent to which expert and consumer perspectives were incorporated into research on population need for substance use services. We were particularly interested in the extent to which consumer perspectives were represented in survey research measuring population need. Because of recent interest in consumer perspectives, due to efforts to improve direct estimation methods that quantify population need for services, [30] we also critically assessed the methodological and measurement approaches used to study consumer-defined need for substance use services and their potential for informing substance use service system planning.

Survey research on population need for substance use services is growing exponentially, with over 600 studies conducted since 2010 alone. However, the imperative to study consumer perspectives–implied both by recognition of the limitations of using diagnostic prevalence to predict service use, and Andersen’s [32] influential model of health services utilization—is belied by the overwhelming emphasis on expert-defined measures designed to quantify need for services in the extant literature. Our review identified relatively few survey research studies assessing population need for substance use services, which incorporated any measure of consumer-defined need. This finding is puzzling, given recognition of the importance of individual perceptions on health in other areas of epidemiology (e.g., the concept of ‘lay epidemiology’; [58, 59]) and social sciences research [60].

Overemphasis on expert conceptions undoubtedly reflects broader challenges of incorporating consumer perspectives into mental health decision-making. Traditionally, psychiatry and other mental health disciplines have granted consumers little-to-no control over mental health services and individual treatment plans [61]. Moreover, those disagreeing with a diagnosis or prescribed treatment were often labeled as in denial, lacking insight, or irrational [62]. Such efforts to defend professional boundaries and legitimate authority have also been observed amongst health researchers [63]. Recent scholarship on consumer engagement describes an epistemological dissonance amongst some researchers, where the value of understanding lay perspectives on health is explicitly endorsed, but ongoing implicit privileging of expertise over experience limits meaningful incorporation of consumer knowledge into scientific findings or related health policy recommendations [63, 64]. This epistemological divide may help explain why despite a strengthening consumer movement which has amplified patient voices and informed mental health care [28, 61, 65], consumer perspectives have yet to exert significant influence over clinical, epidemiological, social science, or health services researchers interested in the substance use treatment gap generally, or in estimating population need for services more specifically.

Another possibility relates to our finding that over half of all survey research on population need for substance use services has been conducted in the United States, where consumer perspectives on need for services may be particularly contentious. In contrast to other jurisdictions, the United States is home to an abundance of private, for-profit managed behavioural health care organizations and has been embroiled in debates over state legislation mandating parity in insurance coverage for medical/surgical and mental health and substance use disorders [11, 24]. Consequently, definitions of ‘need for treatment’ are particularly contested in the United States, and the prospect of incorporating consumer perspectives into service planning (and thereby offering legitimacy to public views on need for care) may raise the specter of out-of-control costs amongst substance use service system researchers, planners, and payers. This theory is supported by the present analysis. In our sample, Canada, Australia, and the United Kingdom accounted for the second, third, and fourth largest numbers of survey research articles, respectively; and all had higher proportions of articles assessing consumer perspectives (24%, 19%, and 17% of articles, respectively) than survey research conducted in the United States (15% of articles).

Studies measuring need for services from a consumer perspective almost always (93%; *n* = 201) reported one or more expert-defined need estimates. Only a minority of those studies (38%; *n* = 61) incorporated more than one consumer-defined need measure, and only two studies (1%) incorporated all three. Very little research in this area was longitudinal or tested hypotheses. These findings suggest that consumer-defined need for substance use services may be conceptually underdeveloped, and more often viewed as a proxy for expert-defined service need, rather than as a construct warranting its own investigation and analysis. Our results also suggest that extant literature in this area has generally avoided efforts to analyze the relationships between perceived need for care, self-assessed barriers to care, and help- seeking from family and friends, and the significance of these relationships for understanding the substance use disorder treatment gap.

Inattention to the conceptual aspects of consumer-defined service need may also account for the disparate way in which perceived need for substance use services, self-assessed barriers to service use, and help - seeking from friends or family are measured in this literature. Many studies used single item measures of unknown psychometric quality. Critically, ‘services’ was inconsistently defined. Some studies asked participants about need for, or barriers to, generic ‘treatment,’ whereas others asked about ‘help’ instead. This is problematic because the notion of ‘treatment’ or ‘help’ may be variously interpreted by different populations. These nonspecific measures may also imply a particular level of intensity (e.g., residential substance use treatment) that participants do not perceive a need for, even if lower intensity or informal supports might be desirable. As a result, estimates of consumer-defined need may vary considerably across studies and populations.

Researchers have developed a range of measurement approaches for assessing patterns of substance use and substance use disorders from an expert perspective. In contrast, less than 10% of studies assessing service need from a consumer perspective used standardized measures. The small proportion of studies that did adopt standardized measures employed several different types of instruments drawn from both general mental health and substance use literatures. Given the availability of an array of instruments, it remains unclear why more research on consumer-defined service need has not employed these, or other standardized measures. Further study is required to investigate the suitability of new and existing standardized instruments for measuring consumer-defined need for substance use services at the population level.

Methodological and measurement weaknesses demonstrated in our review may be attributable to the disciplinary diversity inherent in prioritizing consumer perspectives on population need for substance use services. Research on diagnosis and classification of mental health and substance use disorders in the community is traditionally conducted within the purview of psychiatric epidemiology. However, whereas epidemiologists are concerned with understanding prevalence, correlates, and etiological factors underlying disease, health services researchers are primarily focused on examining system features and other factors that predict service use [66]. Neither of these disciplinary traditions has attended to subjective perceptions with the same enthusiasm as psychosocial research, which focuses on assessing individual perspectives on treatment need in relation to psychosocial factors. Given the diversity of these disciplinary perspectives, the present results suggest that the concept of service need – as defined by consumers of those services – has been neglected in extant literature.

Disciplinary diversity may also explain why so few studies measuring perceived need have used the resulting data to actually estimate system capacity required to meet population need for substance use services. We identified only one such study in our review [57]. Unlike health services research, epidemiological or psychosocial research typically does not prioritize service system planning. While it is unrealistic to expect all survey research to directly inform system planning, it can be argued that research measuring consumer perspectives on service need has the implicit aim of providing relevant data for increasing uptake into care. An outstanding task for health services researchers is to attempt to convert these data into required service system capacity estimates for specific jurisdictions.

### Strengths and limitations of this review

This scoping review offers a critical analysis of 35 years of research on population need for substance use services. It is the first review of its kind to assess the extent to which consumer perspectives are incorporated into this literature, or analyze existing approaches to studying consumer-defined need for substance use services. While our review was comprehensive, we focused on peer-reviewed, English literature and thus inevitably missed studies published in other languages and possibly in the grey literature. We also limited our scope to substance use disorders and did not assess studies focused on general mental health. This decision is justifiable, as many jurisdictions plan substance use services separately from other mental healthcare, but it limits the applicability of our results to services for substance use problems. We coded for the presence of both expert and consumer-defined measures of need, but we did not assess the quality of this evidence, nor characterize how these variables were treated in each study. We also provide no synthesis of the comprehensive empirical findings of consumer-defined need studies in this area. Future systematic reviews are needed to analyze whether consumer perspectives overestimate or underestimate population need for substance use services, how these estimates may vary by sociodemographic and cultural characteristics, and the relative weight that should be given to consumer perspectives *vis*-*a*-*vis* objective perspectives in treatment system capacity estimates. EH conducted the full-text screen of all included articles independently, which may have introduced some bias. We attempted to mitigate this bias by ensuring other authors were consulted and a consensus was reached for ambiguous cases.

## Conclusion

A large volume of research over the past 35 years has addressed population need for substance use services, and scholarship in this area has grown exponentially over the past decade. In the US and elsewhere, there has been a growing receptivity to consumer voices in mental healthcare practice and policymaking, enabling consumers to play a larger role in their own treatment decisions and to advocate collectively to improve services [28]. Yet despite growth in research on population need for substance use services, and mainstreaming of consumer perspectives in some aspects of mental healthcare, research in this area generally downplays consumer perspectives, both in quantifying population need for services, and in actual service system planning. This knowledge gap challenges our ability to predict service utilization and improve uptake into substance use services. Meaningful incorporation of consumer perspectives into research on population need for substance use services is overdue, and we have identified several ways to address this task. Concerted interdisciplinary research efforts are needed to refine the concept of consumer-defined need, examine relationships between its constituent constructs, and psychometrically validate and standardize new or adapted measurement instruments. Future health services research should also evaluate approaches for incorporating consumer-defined need measures to enhance estimates of required service system capacity. In the absence of these efforts, the potential for consumer-defined measures of need to provide further insight into the substance use disorder treatment gap, and mitigate morbidity and mortality of substance misuse, will remain unrealized.

## Additional files

Additional file 1: Database-specific controlled vocabularies. Provides all database-specific controlled vocabularies used in the systematic search process. (DOCX 126 kb)
Additional file 2: Data extraction framework. Provides the framework used to extract data from articles included in this review. (DOCX 99 kb)
Additional file 3: Consumer-defined need studies. All studies assessing consumer perspective on need for substance use services identified through systematic search (*n* = 217). (DOCX 52 kb)
