# Supplementary material for: The role of consumer perspectives in estimating population need for substance use services: a scoping review
Source: BMC Health Serv Res. 2017 Mar 20;17:217. doi: 10.1186/s12913-017-2153-z (PMC5359989; doi:10.1186/s12913-017-2153-z)
Supplement: Additional file 1: — Database-specific controlled vocabularies. Provides all database-specific controlled vocabularies used in the systematic search process. (DOCX 126 kb) [file 12913_2017_2153_MOESM1_ESM.docx]

## Additional File 1: Database-specific controlled vocabularies

**Ovid MEDLINE® In-Process & Other Non-Indexed Citations, Ovid MEDLINE® Daily and Ovid MEDLINE®**

1. substance-related disorders/ or exp alcohol-related disorders/ or amphetamine-related disorders/ or cocaine-related disorders/ or drug overdose/ or inhalant abuse/ or marijuana abuse/ or exp opioid-related disorders/ or phencyclidine abuse/ or psychoses, substance-induced/ or substance abuse, intravenous/ or exp substance withdrawal syndrome/ or exp street drugs/

2. ((substance or drug* or alcohol* or cocaine or meth or methamphetamine or amphetamine or crack or heroin or opiate* or opioid* or narcotic* or morphine or mari#uana or cannabis or hash* or lsd or hallucinogen* or inhalant* or oxycodon* or vicodin or codeine or fentanyl) adj3 (abus* or addict* or dependen* or misus* or withdrawal or overdose* or detox* or user*)).ti,kf.

3. (illicit drug* or illicit substance* or illegal drug* or illegal substance* or street drug* or needle exchange* or syringe exchange or safe injection or supervised injection).ti,kf.

4. 1 or 2 or 3

5. *"health services needs and demand"/ or *needs assessment/ or *Health Services Accessibility/

6. (need or needs or barrier*).ti.

7. ((service* or resource* or care or treatment or facilit* or hospital* or rehab* or harm reduction) adj3 (need or needs or access* or demand* or availab* or barrier*)).ab,kf.

8. ((unmet or perceive*) adj2 need*1).ab,kf.

9. *Health Resources/ or *Resource Allocation/

10. *Health Planning/ or exp *Regional Health Planning/

11. planning.ti. or ((plan* or allocat*) adj5 (service* or resource* or facilit*)).ab,kf.

12. or/5-11

13. 4 and 12

14. (ep or sd or td or ut or ec or og).fs.

15. *forecasting/ or models, statistical/ or models, theoretical/ or epidemologic factors/ or linear models/ or logistic models/ or (linear regression or capture recapture).ti,ab,kf.

16. regression analysis/ or (logistic regression or regression analysis or representative sampl* or prevalence or survellance).ti,ab,kf.

17. Health Surveys/ or Questionnaires/

18. (national or global or regional or state wide or statewide or province wide or nationwide or nation wide or country wide or countrywide).ti,ab,kf.

19. Population Surveillance/ or Population/

20. Demography/ or (demograph* or sociodemograph*).ti,ab,kf.

21. data collection/

22. ("National Epidemiologic Survey on Alcohol and Related Conditions" or nesarc or "National Survey on Drug Use and Health" or nsduh or NLAES or "National Drug and Alcoholism Treatment Utilization Survey" or NDATUS or global burden or burden of disease).ti,ab,kf.

23. (longitudinal or prospective or retrospective or cross sectional or cohort or algorithm* or methodolog* or multivaria* or predictor* or indicator* or odds).mp.

24. model*.ti.

25. or/14-24

26. 13 and 25

27. (estimat* and (prevalence or acces* or utili* or population or service*)).ti.

28. (estimat* adj3 (prevalence or acces* or utili* or population or service*)).ab,kf.

29. 27 or 28

30. 4 and 29

31. 26 or 30

32. limit 31 to (english language and yr="1980 -Current")

33. remove duplicates from 32

**Ovid EMBASE**

1. addiction/ or alcoholism/ or withdrawal syndrome/

2. exp drug dependence/ or "drug dependence treatment"/

3. street drug/

4. ((substance or drug* or alcohol* or cocaine or meth or methamphetamine or amphetamine or crack or heroin or opiate* or opioid* or narcotic* or morphine or mari#uana or cannabis or hash* or lsd or hallucinogen* or inhalant* or oxycodon* or vicodin or codeine or fentanyl) adj3 (abus* or addict* or dependen* or misus* or withdrawal or overdose* or detox* or user*)).ti.

5. (illicit drug* or illicit substance* or illegal drug* or illegal substance* or street drug* or needle exchange* or syringe exchange or safe injection or supervised injection).ti.

6. or/1-5

7. needs assessment/ or "health care need"/ or health care access/ or health care availability/

8. (needs or needs or barrier*).ti.

9. ((service* or resource* or care or treatment or facilit* or hospital* or rehab* or harm reduction) adj3 (need or needs or access* or demand* or availab* or barrier*)).ab.

10. ((unmet or perceive*) adj2 need*1).ab.

11. health care planning/

12. resource allocation/

13. planning.ti. or ((plan* or allocat*) adj5 (service* or resource* or facilit*)).ab.

14. or/7-13

15. 6 and 14

16. "prediction and forecasting"/ or computer prediction/ or forecasting/ or prediction/

17. "population and population related phenomena"/ or population/ or population model/ or population research/ or population risk/ or rural population/ or suburban population/ or susceptible population/ or urban population/ or vulnerable population/

18. process model/ or mathematical model/ or statistical model/ or theoretical model/

19. exp regression analysis/

20. (logistic regression or regression analysis or representative sampl* or prevalence or survellance).ti,ab.

21. health survey/

22. exp questionnaire/

23. data collection method/ or questionnaire/

24. demography/

25. (demograph* or sociodemograph*).ti,ab.

26. ("National Epidemiologic Survey on Alcohol and Related Conditions" or nesarc or "National Survey on Drug Use and Health" or nsduh or NLAES or "National Drug and Alcoholism Treatment Utilization Survey" or NDATUS or global burden or burden of disease).ti,ab.

27. (longitudinal or prospective or retrospective or cross sectional or cohort or algorithm* or methodolog* or multivaria* or predictor* or indicator* or odds).ti,ab.

28. (national or global or regional or state wide or statewide or province wide or nationwide or nation wide or country wide or countrywide).ti,ab.

29. model*.ti.

30. or/16-29

31. 15 and 30

32. (estimat* and (prevalence or acces* or utili* or population or service*)).ti.

33. (estimat* adj3 (prevalence or acces* or utili* or population or service*)).ab.

34. 32 or 33

35. 6 and 34

36. 31 or 35

37. limit 36 to (english language and yr="1980 -Current")

38. remove duplicates from 37

**Ovid PsycInfo**

1. exp drug abuse/

2. exp drug dependency/

3. addiction/ or exp alcoholism/ or exp drug addiction/

4. drug withdrawal/ or alcohol withdrawal/ or detoxification/

5. drug rehabilitation/ or exp alcohol rehabilitation/ or sobriety/ or exp twelve step programs/

6. ((substance or drug* or alcohol* or cocaine or meth or methamphetamine or amphetamine or crack or heroin or opiate* or opioid* or narcotic* or morphine or mari#uana or cannabis or hash* or nicotine or lsd or hallucinogen* or inhalant* or oxycodon* or vicodin or codeine or fentanyl) adj3 (abus* or addict* or dependen* or misus* or withdrawal or overdose* or detox* or user*)).ti,ab,id.

7. (illicit drug* or illicit substance* or illegal drug* or illegal substance* or street drug* or needle exchange* or syringe exchange or safe injection or supervised injection).ti,ab,id.

8. "Drug & Alcohol Rehabilitation ".cc.

9. "Substance Abuse & Addiction ".cc.

10. or/1-9

11. needs assessment/ or health service needs/ or utilization reviews/

12. (need or needs or barrier*).ti.

13. ((service* or resource* or care or treatment or facilit* or hospital* or rehab* or harm reduction) adj3 (need or needs or access* or demand* or availab* or barrier*)).ab,id.

14. ((unmet or perceive*) adj2 need*1).ab,id.

15. exp Health Care Utilization/

16. health care services/ or health care seeking behavior/ or help seeking behavior/

17. resource allocation/

18. planning.ti. or ((plan* or allocat*) adj5 (service* or resource* or facilit*)).ab,id.

19. or/11-18

20. 10 and 19

21. prediction/

22. exp estimation/

23. models/

24. population/ or "population (statistics)"/ or demographic characteristics/

25. surveys/ or data collection/ or questionnaires/

26. exp multivariate analysis/

27. exp statistical regression/

28. (national or global or regional or state wide or statewide or province wide or nationwide or nation wide or country wide or countrywide).ti,ab,id.

29. (logistic regression or regression analysis or representative sampl* or prevalence or survellance or linear regression or capture recapture).ti,ab,id.

30. (demograph* or sociodemograph*).ti,ab,id.

31. ("National Epidemiologic Survey on Alcohol and Related Conditions" or nesarc or "National Survey on Drug Use and Health" or nsduh or NLAES or "National Drug and Alcoholism Treatment Utilization Survey" or NDATUS or global burden or burden of disease).ti,ab,id.

32. (longitudinal or cross sectional or cohort or algorithm* or methodolog* or multivaria* or predictor* or indicator* or odds).mp.

33. (model* or estimat*).ti.

34. or/21-33

35. 20 and 34

36. limit 35 to (english language and yr="1980 -Current")

37. remove duplicates from 36

**Ovid All EBM [Evidence-Based Medicine] Reviews (Cochrane Database of Systematic Reviews, Cochrane Central Register of Controlled Trials, Cochrane Methodology Register, Database of Abstracts of Reviews of Effect, Health Technology Assessment Database and Economic Evaluation Database)**

1. substance-related disorders/ or exp alcohol-related disorders/ or amphetamine-related disorders/ or cocaine-related disorders/ or drug overdose/ or inhalant abuse/ or marijuana abuse/ or exp opioid-related disorders/ or phencyclidine abuse/ or psychoses, substance-induced/ or substance abuse, intravenous/ or exp substance withdrawal syndrome/ or exp street drugs/

2. ((substance or drug* or alcohol* or cocaine or meth or methamphetamine or amphetamine or crack or heroin or opiate* or opioid* or narcotic* or morphine or mari#uana or cannabis or hash* or lsd or hallucinogen* or inhalant* or oxycodon* or vicodin or codeine or fentanyl) adj3 (abus* or addict* or dependen* or misus* or withdrawal or overdose* or detox* or user*)).ti,ab,kf.

3. (illicit drug* or illicit substance* or illegal drug* or illegal substance* or street drug* or needle exchange* or syringe exchange or safe injection or supervised injection).ti,ab,kf.

4. 1 or 2 or 3

5. "health services needs and demand"/ or needs assessment/ or Health Services Accessibility/

6. (need or needs or barrier*).ti.

7. ((service* or resource* or care or treatment or facilit* or hospital* or rehab* or harm reduction) adj3 (need or needs or access* or demand* or availab* or barrier*)).ab,kf.

8. ((unmet or perceive*) adj2 need*1).ab,kf.

9. Health Resources/ or Resource Allocation/

10. Health Planning/ or exp Regional Health Planning/

11. planning.ti. or ((plan* or allocat*) adj5 (service* or resource* or facilit*)).ab,kf.

12. or/5-11

13. 4 and 12

14. (ep or sd or td or ut or ec or og).fs.

15. forecasting/ or models, statistical/ or models, theoretical/ or epidemologic factors/ or linear models/ or logistic models/ or (linear regression or capture recapture).ti,ab,kf.

16. regression analysis/ or (logistic regression or regression analysis or representative sampl* or prevalence or survellance).ti,ab,kf.

17. Health Surveys/ or Questionnaires/

18. (national or global or regional or state wide or statewide or province wide or nationwide or nation wide or country wide or countrywide).ti,ab,kf.

19. Population Surveillance/ or Population/

20. Demography/ or (demograph* or sociodemograph*).ti,ab,kf.

21. data collection/

22. ("National Epidemiologic Survey on Alcohol and Related Conditions" or nesarc or "National Survey on Drug Use and Health" or nsduh or NLAES or "National Drug and Alcoholism Treatment Utilization Survey" or NDATUS or global burden or burden of disease).ti,ab,kf.

23. (longitudinal or prospective or retrospective or cross sectional or cohort or algorithm* or methodolog* or multivaria* or predictor* or indicator* or odds).mp.

24. model*.ti.

25. or/14-24

26. 13 and 25

27. (estimat* and (prevalence or acces* or utili* or population or service*)).ti.

28. (estimat* adj3 (prevalence or acces* or utili* or population or service*)).ab,kf.

29. 27 or 28

30. 4 and 29

31. 26 or 30

32. limit 31 to (english language and yr="1980 -Current")

33. remove duplicates from 32

**EBSCO CINAHL Plus with Full-text**

S1 (MH "Substance Use Disorders") OR (MH "Alcohol Withdrawal Syndrome+") OR (MH "Substance Withdrawal Syndrome+") OR (MH "Substance Dependence+") OR (MH "Substance Abuse, Intravenous") OR (MH "Inhalant Abuse") OR (MH "Alcohol-Related Disorders+") OR (MH "Substance Abuse") or ( (substance or drug* or alcohol* or cocaine or meth or methamphetamine or amphetamine or crack or heroin or opiate* or opioid* or narcotic* or morphine or marijuana or marihuana or cannabis or hash* or lsd or hallucinogen* or inhalant* or oxycodon* or vicodin or codeine or fentanyl) n3 (abus* or addict* or dependen* or misus* or withdrawal or overdose* or detox* or user*) ) OR ( "illicit drug*" or "illicit substance*" or "illegal drug*" or "illegal substance*" or "street drug*" or "needle exchange*" or "syringe exchange" or "safe injection" or "supervised injection" )

S2 (MH "Health Services Needs and Demand+") OR (MH "Needs Assessment") OR (MH "Health Services Accessibility+") OR (MH "Health Resource Utilization") OR (MH "Health Resource Allocation") OR (MH "Health and Welfare Planning") OR (MH "Health Facility Planning") OR (MH "National Health Programs") OR (MH "State Health Plans") OR (MH "Strategic Planning+") OR TI ( need or needs or barrier* or planning ) OR ( (service* or resource* or care or treatment or facilit* or hospital* or rehab* or "harm reduction") n3 (need or needs or access* or demand* or availab* or barrier*) ) OR ( (unmet or perceive*) n2 (need or needs) ) OR ( (plan* or allocat*) n5 (service* or resource* or facilit*) )

S3 S1 AND S2

S4 (MH "Forecasting") OR (MH "Models, Statistical") OR (MH "Models, Theoretical") OR (MH "Regression+") OR (MH "Surveys") OR (MH "Population Surveillance") OR (MH "Questionnaires") OR (MH "Structured Questionnaires") OR (MH "Demography") OR (MH "Geographic Factors") OR (MH "Population+") OR (MH "Data Collection") OR ( regression or "capture recapture" or "representative sampl*" or prevalence or survellance ) OR ( national or global or regional or "state wide" or statewide or "province wide" or nationwide or "nation wide" or "country wide" or countrywide or demograph* or sociodemograph* ) OR ( "National Epidemiologic Survey on Alcohol and Related Conditions" or nesarc or "National Survey on Drug Use and Health" or nsduh or NLAES or "National Drug and Alcoholism Treatment Utilization Survey" or NDATUS or "global burden" or "burden of disease" ) OR ( longitudinal or prospective or retrospective or cross sectional or cohort or algorithm* or methodolog* or multivaria* or predictor* or indicator* or odds ) OR TI model*

S5 S3 AND S4

S6 TI ( estimat* and (prevalence or acces* or utili* or population or service*) ) OR AB ( estimat* n3 (prevalence or acces* or utili* or population or service*) )

S7 S1 AND S6

S8 S5 OR S7

Limiters - Published Date: 19800101-20151231

**Scopus**

( TITLE ( ( substance OR drug* OR alcohol* ) AND ( abus* OR addict* OR dependen* OR misus* OR withdrawal OR overdose* OR detox* OR user* ) ) OR TITLE ( cocaine OR meth OR methamphetamine OR amphetamine OR crack OR heroin OR opiate* OR opioid* OR narcotic* OR morphine OR marijuana OR marihuana OR cannabis OR hash* OR lsd OR hallucinogen* OR inhalant* OR oxycodon* OR vicodin OR codeine OR fentanyl OR "illicit drug*" OR "illicit substance*" OR "illegal drug*" OR "illegal substance*" OR "street drug*" OR "needle exchange*" OR "syringe exchange" OR "safe injection" OR "supervised injection" ) ) AND ( TITLE ( need OR needs OR barrier* OR planning ) OR TITLE-ABS-KEY ( ( service W/3 ( need OR needs OR access* OR demand* OR availab* OR barrier* ) ) OR ( resource* W/3 ( need OR needs OR access* OR demand* OR availab* OR barrier* ) ) OR ( care W/3 ( need OR needs OR access* OR demand* OR availab* OR barrier* ) ) OR ( treatment W/3 ( need OR needs OR access* OR demand* OR availab* OR barrier* ) ) OR ( facilit* W/3 ( need OR needs OR access* OR demand* OR availab* OR barrier* ) ) OR ( hospital* W/3 ( need OR needs OR access* OR demand* OR availab* OR barrier* ) ) ) OR TITLE-ABS-KEY ( ( unmet W/2 ( need OR needs ) ) OR ( perceive* W/2 ( need OR needs ) ) OR ( plan* W/5 ( service* OR resource* OR facilit* ) ) OR ( "harm reduction" W/3 ( need OR needs OR access* OR demand* OR availab* OR barrier* ) ) OR ( allocat* W/5 ( service* OR resource* OR facilit* ) ) ) ) AND ( TITLE-ABS-KEY ( forecast* OR regression OR population OR questionnaire* OR demograph* OR sociodemograph* OR regression OR "capture recapture" OR "representative sampl*" OR prevalence OR survellance OR national OR global OR regional OR "state wide" OR statewide OR "province wide" OR nationwide OR "nation wide" OR "country wide" OR countrywide OR "National Epidemiologic Survey on Alcohol and Related Conditions" OR nesarc OR "National Survey on Drug Use and Health" OR nsduh OR nlaes OR "National Drug and Alcoholism Treatment Utilization Survey" OR ndatus OR "global burden" OR "burden of disease" OR longitudinal OR prospective OR retrospective OR cross sectional OR cohort OR algorithm* OR methodolog* OR multivaria* OR predictor* OR indicator* OR odds ) OR TITLE ( model* ) ) OR ( TITLE-ABS-KEY ( substance OR drug* OR alcohol* OR cocaine OR meth OR methamphetamine OR amphetamine OR crack OR heroin OR opiate* OR opioid* OR narcotic* OR morphine OR marijuana OR marihuana OR cannabis OR hash* OR lsd OR hallucinogen* OR inhalant* OR oxycodon* OR vicodin OR codeine OR fentanyl ) AND TITLE-ABS-KEY ( abus* OR addict* OR dependen* OR misus* OR withdrawal OR overdose* OR detox* OR user* ) OR TITLE ( "illicit drug*" OR "illicit substance*" OR "illegal drug*" OR "illegal substance*" OR "street drug*" OR "needle exchange*" OR "syringe exchange" OR "safe injection" OR "supervised injection" ) AND TITLE ( estimat* AND ( prevalence OR acces* OR utili* OR population OR service* ) ) OR ABS ( estimat* W/3 ( prevalence OR acces* OR utili* OR population OR service* ) ) ) AND ( LIMIT-TO ( PUBYEAR , 2015 ) OR LIMIT-TO ( PUBYEAR , 2014 ) OR LIMIT-TO ( PUBYEAR , 2013 ) OR LIMIT-TO ( PUBYEAR , 2012 ) OR LIMIT-TO ( PUBYEAR , 2011 ) OR LIMIT-TO ( PUBYEAR , 2010 ) OR LIMIT-TO ( PUBYEAR , 2009 ) OR LIMIT-TO ( PUBYEAR , 2008 ) OR LIMIT-TO ( PUBYEAR , 2007 ) OR LIMIT-TO ( PUBYEAR , 2006 ) OR LIMIT-TO ( PUBYEAR , 2005 ) OR LIMIT-TO ( PUBYEAR , 2004 ) OR LIMIT-TO ( PUBYEAR , 2003 ) OR LIMIT-TO ( PUBYEAR , 2002 ) OR LIMIT-TO ( PUBYEAR , 2001 ) OR LIMIT-TO ( PUBYEAR , 2000 ) OR LIMIT-TO ( PUBYEAR , 1999 ) OR LIMIT-TO ( PUBYEAR , 1998 ) OR LIMIT-TO ( PUBYEAR , 1997 ) OR LIMIT-TO ( PUBYEAR , 1996 ) OR LIMIT-TO ( PUBYEAR , 1995 ) OR LIMIT-TO ( PUBYEAR , 1994 ) OR LIMIT-TO ( PUBYEAR , 1993 ) OR LIMIT-TO ( PUBYEAR , 1992 ) OR LIMIT-TO ( PUBYEAR , 1991 ) OR LIMIT-TO ( PUBYEAR , 1990 ) OR LIMIT-TO ( PUBYEAR , 1988 ) OR LIMIT-TO ( PUBYEAR , 1987 ) OR LIMIT-TO ( PUBYEAR , 1986 ) OR LIMIT-TO ( PUBYEAR , 1985 ) OR LIMIT-TO ( PUBYEAR , 1984 ) OR LIMIT-TO ( PUBYEAR , 1980 ) )

**Web of Science Core Collection**

#1 TI=((substance or drug* or alcohol* or cocaine or meth or methamphetamine or amphetamine or crack or heroin or opiate* or opioid* or narcotic* or morphine or marijuana or marihuana or cannabis or hash* or lsd or hallucinogen* or inhalant* or oxycodon* or vicodin or codeine or fentanyl) AND (abus* or addict* or dependen* or misus* or withdrawal or overdose* or detox* or user*) or "illicit drug*" or "illicit substance*" or "illegal drug*" or "illegal substance*" or "street drug*" or "needle exchange*" or "syringe exchange" or "safe injection" or "supervised injection")

#2 TI=( need or needs or barrier* or planning ) OR TS=( (service* or resource* or care or treatment or facilit* or hospital* or rehab* or “harm reduction”) near/3 (need or needs or access* or demand* or availab* or barrier*) OR (unmet or perceive*) near/2 (need or needs) OR (plan* or allocat*) near/5 (service* or resource* or facilit*) )

#3 #1 AND #2

#4 TS=(forecast* or regression or population or questionnaire* or demograph* or sociodemograph* or regression or "capture recapture" or "representative sampl*" or prevalence or survellance or national or global or regional or "state wide" or statewide or "province wide" or nationwide or "nation wide" or "country wide" or countrywide or "National Epidemiologic Survey on Alcohol and Related Conditions" or nesarc or "National Survey on Drug Use and Health" or nsduh or NLAES or "National Drug and Alcoholism Treatment Utilization Survey" or NDATUS or "global burden" or "burden of disease" or longitudinal or prospective or retrospective or cross sectional or cohort or algorithm* or methodolog* or multivaria* or predictor* or indicator* or odds) or TI=(model*)

#5 #3 AND #4

#6 TS=( estimat* near/3 (prevalence or acces* or utili* or population or service*) )

#7 #1 AND #6

#8 #5 OR #7
