# Supplementary material for: The role of consumer perspectives in estimating population need for substance use services: a scoping review
Source: BMC Health Serv Res. 2017 Mar 20;17:217. doi: 10.1186/s12913-017-2153-z (PMC5359989; doi:10.1186/s12913-017-2153-z)
Supplement: Additional file 2: — Data extraction framework. Provides the framework used to extract data from articles included in this review. (DOCX 99 kb) [file 12913_2017_2153_MOESM2_ESM.docx]

## Additional File 2. Data extraction framework

| **All studies** | | | | |
| --- | --- | --- | --- | --- |
| **Year** | **Year Published** | Year the study/article was published | Format = YYYY |  |
| **Study Location** | **Single Country** | Is the data presented in the study from only one country? For non-empirical articles, treat as single country and specify the home country of the lead author in the next field. | 0 = No 1 = Yes |  |
|  | **Specify Country** | Specify either the single country in which the data were collected, or the home country of the lead author for non-empirical articles. Code the country's global region in the next field | String variable- write in country name |  |
|  | **Country classification** | Specify whether the single country is high income, or low-middle income | 1 = High income  2 = Low income | Determined according to World Bank classification system http://data.worldbank.org/about/country-and-lending-groups |
|  | **Specify Country Region** | Code the global region of the single country where the data were collected | 1 = North America (Canada & US) 2 = Europe 3 = Australia and Oceania 4 = Latin America and Caribbean 5 = Africa 6 = Middle East 7 = Asia | 1. North America = Canada and US (including Puerto Rico)  2. Europe = all countries in the European Union and Albania, Andorra, Belarus, Bosnia, Croatia, Faroe Islands, Gibraltar, Guerney and Alderney, Iceland, Jersey, Kosovo, Liechtenstein, Macedonia, Man, Island of, Moldova, Monaco, Montenegro, Norway, Russia, San Marino, Serbia, Svalbard and Jan Mayan Islands, Switzerland, Turkey, Ukraine, Vatican City State (Holy See).  3. Australia and Oceania Australia = Fiji, French Polynesia, Guam, Kiribati, Marshall Islands, Micronesia, New Caledonia, New Zealand, Papua New Guinea, Samoa, Samoa (American), Solomon Islands, Tonga, Vanuatu  4. Latin America and Caribbean = Mexico, South America, Central America, and Caribbean countries  5. Africa = All countries on the African continent  6. Middle East = Bahrain, Iraq, Iran, Israel, Jordan, Kuwait, Lebanon, Oman, Palestine, Qatar, Saudi Arabia, Syria, United Arab Emirates, Yemen  7. Asia = Afghanistan, Armenia, Azerbaijan, Bangladesh, Bhutan, Brunei Darussalam, Cambodia, China, Georgia, Hong Kong, India, Indonesia, Japan, Kazakhstan, North Korea, South Korea, Kyrgyzstan, Laos, Macao, Malaysia, Maldives, Mongolia, Myanmar (ex-Burma), Nepal, Pakistan, Philippines, Singapore, Sri Lanka (ex-Ceilan), Taiwan, Tajikistan, Thailand, Timor Leste (West), Turkmenistan, Uzbekistan, Vietnam  8. Multiple regions = data are from 2 or more of the regions listed above |
|  | **Multiple Countries** | Is the data presented in the study from more than one country? [Not applicable for non-empirical articles] Specify region (rather than countries) in the next field. | 0 = No  1 = Yes | For studies where data were collected in more than one country |
|  | **Specify Multiple Countries' Region** | Code the global region where the data were collected, or select 'multiple regions' if the data were collected from country’s spanning more than one. This field is only for studies where data were collected in multiple countries. | 1 = North America (Canada & US) 2 = Europe 3 = Australia and Oceania 4 = Latin America and Caribbean 5 = Africa 6 = Middle East 7 = Asia 8 = Multiple regions (two or more of the regions listed above) | 1. North America = Canada and US  2. Europe = all countries in the European Union and Albania, Andorra, Belarus, Bosnia, Croatia, Faroe Islands, Gibraltar, Guerney and Alderney, Iceland, Jersey, Kosovo, Liechtenstein, Macedonia, Man, Island of, Moldova, Monaco, Montenegro, Norway, Russia, San Marino, Serbia, Svalbard and Jan Mayen Islands, Switzerland, Turkey, Ukraine, Vatican City State (Holy See).  3. Australia and Oceania Australia = Fiji, French Polynesia, Guam, Kiribati, Marshall Islands, Micronesia, New Caledonia, New Zealand, Papua New Guinea, Samoa, Samoa (American), Solomon Islands, Tonga, Vanuatu  4. Latin America and Caribbean = Mexico, South America, Central America, and Caribbean countries  5. Africa = All countries on the African continent  6. Middle East = Bahrain, Iraq, Iran, Israel, Jordan, Kuwait, Lebanon, Oman, Palestine, Qatar, Saudi Arabia, Syria, United Arab Emirates, Yemen  7. Asia = Afghanistan, Armenia, Azerbaijan, Bangladesh, Bhutan, Brunei Darussalam, Cambodia, China, Georgia, Hong Kong, India, Indonesia, Japan, Kazakhstan, North Korea, South Korea, Kyrgyzstan, Laos, Macao, Malaysia, Maldives, Mongolia, Myanmar (ex-Burma), Nepal, Pakistan, Philippines, Singapore, Sri Lanka (ex-Ceilan), Taiwan, Tajikistan, Thailand, Timor Leste (West), Turkmenistan, Uzbekistan, Vietnam  8. Multiple regions = data are from 2 or more of the regions listed above |
| **Paper type** | **Empirical paper** | Is the article empirical or non-empirical? | 0 = No  1 = Yes | Non-empirical: Does not present any empirical (quantitative or qualitative) data. Includes articles such as systematic or non-systematic literature reviews (no data analyzed), methods papers (where no data are presented), etc.  Empirical: Paper presents quantitative or qualitative data. Includes articles such as quantitative, qualitative or mixed methods studies; systematic reviews; modelling studies; etc. |
| **Data sources** | **Biological Data** | Empirical studies only: Does the paper present biological measures of substance use? | 0 = No  1 = Yes | Includes articles that present biological data on substance use via individual or aggregate biological samples. E.g., hair analysis, wastewater analysis, urinalysis, etc.  **Not mutually exclusive category (e.g., empirical studies can have more than one data source) |
|  | **Administrative Data** | Empirical studies only: Does the paper analyze/present any administrative data? | 0 = No 1 = Yes | Census, area alcohol sales, treatment registers, arrest records, hospital admissions, care episodes, birth registers, Medicare registers, death records, etc.  **Not mutually exclusive category (e.g., empirical studies can have more than one data source) |
|  | **Qualitative Data** | Empirical studies only: Does the article analyze/present any qualitative data? | 0 = No 1 = Yes | Includes empirical studies, which present qualitative data (alone or alongside quantitative data).  **Not mutually exclusive category (e.g., empirical studies can have more than one data source) |
|  | **Population survey Data** | Empirical studies only: Does the article analyze/present any population survey research data? | 0 = No 1 = Yes | Uses data from direct contact self-reports to estimate population need for services directly.  **Not mutually exclusive category (e.g., empirical studies can have more than one data source) |
| **Sampling** | **Substance users only** | Survey research studies: Did the survey research study only recruit substance users? | 0 = No 1 = Yes | Includes survey research studies in which eligibility was (past month, past year, and/or lifetime) substance use. This includes studies, which targeted recruitment to people with alcohol and/or substance use disorders. |
| **Objective need** | **Objective need measured** | The survey research examines objective need for substance use services | 0 = No 1 = Yes | The survey research study examines objective (expert-defined) need for substance use services. This means assessment of need for services is based on expert (rather than consumer/patient/participant) judgement.   Objective need studies contain at least one of the following measures: 1. Substance use prevalence  2. Substance use disorder prevalence 3. Self-reported or objective rates of primary, general, or specialty service utilization 4. Self-reported or objective rates of formal help seeking (e.g., accessing care from primary, generalist, or specialist [health or non-health] professionals).  ** Not mutually exclusive (study can examine both objective and perceived need) |
| **Objective need variables** | **Substance use prevalence** | Survey research studies: Does the survey research paper report a substance use prevalence estimate? | 0 = No 1 = Yes | The survey research provides a substance use prevalence estimate (e.g., % of people in the general or special population reported using one or more licit or illicit substances).  [Does not include articles in which entire sample is comprised of substance users]  **not mutually exclusive category (e.g., population survey papers can measure more than one need variable. |
|  | **Substance use disorder prevalence** | Survey research studies: Does the survey research report a substance use disorder prevalence estimate? | 0 = No 1 = Yes | The survey research provides an (objective, expert-derived) substance use disorder prevalence estimate. Includes estimates of the proportion of the sample or population who are experiencing: substance dependence, substance abuse, substance use disorder, or addiction. [Includes: binge-drinking; heavy smoking; alcohol problems as these patterns of use may indicate need for substance use services].  **Not mutually exclusive category (e.g., population survey papers can measure more than one need variable. |
|  | **Substance use service utilization** | Does the survey research report rates of substance use or mental health service use? [One or more general or specialty services; includes formal help-seeking] | 0 = No 1 = Yes | The survey research provides an estimate of the proportion of the sample that used primary care, general, or specialty services for help with substance use and/or mental health problems. This includes formal help seeking.  **Not mutually exclusive category (e.g., population survey papers can measure more than one objective need variable. |
| **Subjective need measurement** | **Subjective need measure** | The survey research incorporates a measure of subjective need for substance use services | 0 = No 1 = Yes | The survey research study examines subjective need for substance use services.   Consumer perspective studies contain at least one of the following measures: 1. Perceived need or perceived unmet need for care (for substance use or mental health problems)  2. Self-reported rates of help seeking from social networks, friends and family 3. Self-assessed barriers to care.  ** Not mutually exclusive (study can examine both objective and subjective need)  excludes treatment readiness, hypothetical studies (e.g., if you need treatment would you go to...). Also excludes studies which state that pn was measured but, do not present data from these measures. Examples of pn instruments: Camberwell Assessment of Need, Cardinal Needs Schedule |
| **Subjective need studies** | | | | |
| **Study Design** | **Cross-sectional design** | Does the study report data from a single cross-sectional or multiple cross-sectional research project? | 0 = No 1 = Yes, single cross sectional;  2 = Yes, multiple cross sectional | Cross-sectional: Survey data reported in the study were captured during only one time period, with no follow-up of participants.  Multiple cross-sectional: Survey data reported in the study were captured during multiple time periods, but amongst different groups of participants (e.g., participants not followed over time). |
|  | **Longitudinal design** | Does the study report longitudinal data? | 0 = No 1 = Yes | Longitudinal: Survey data reported in the study were captured during multiple time periods, following the same participants over time. |
| **Sample characteristics** | **Size of (community-based) sample** | How many participants were included in the study? | Enter number (round to whole number) -9 = mean age unavailable | Aggregate of all samples in multi-site studies   Report sample size for community-based proportion only, when available. If not available, report for entire sample.   *Report N when available for general population studies* |
|  | **Sex of (community-based) participants** | Did the study include males, females or both? | 0 = Male 1 = Female 2 = Both males and females | Did the study include males, females, or both? If sex is not reported, use gender. If gender is not reported either, code as unavailable.  Report demographics for community-based proportion only, when available. If not available, report for entire sample. |
|  | **Mean age of (community-based) participants** | What was the mean age of participants reported for the single sample, or across multiple samples? | Enter number (round to whole number) | Specify the mean age of participants, rounded to the nearest whole number (e.g., no decimal points). If multiple datasets are analyzed, code the total mean age across datasets (may need to calculate).  Report demographics for community-based proportion only, when available. If not available, report for entire sample. |
| **Target population** | **General adult population** | Does the survey target the general adult population? | 0 = No 1 = Yes | Articles that estimate need for services amongst general adult population (e.g., all adults in one city, state, region, country, etc.) |
|  | **Specialty population** | Does the survey target a specific specialty population? | 0 = No 1 = Yes | Articles that estimate need for services amongst specific special population (e.g., college students, seniors, military personnel, cultural minority groups, etc.) |
|  | **Specify the specialty population** | Specify the specialty population as described by the authors | Write in description; string variable | E.g., opioid users; crack cocaine smokers, college students, secondary school students, farm workers, etc. |
| **Subjective need measures** | **Perceived need** | Does the study estimate whether all or a proportion of the sample or population perceived a need for care for substance use or mental health problems (whether or not this need was met or unmet)?  Specify type of measurement in fields below | 0 = No 1 = Yes | Does the study estimate the proportion of the sample or population who perceived a need for care for substance use or mental health problems?  Includes attempts to measure either perceived need or perceived unmet need for treatment and/or non-treatment seekers in community based samples  **not mutually exclusive, e.g., study can estimate more than one perceived need-related variable |
|  | **Single Item Perceived Need** | For studies, which measure perceived need: Is this variable measured using a single item measure? | 0 = No 1 = Yes | E.g., uses one question to measure perceived need  Not mutually exclusive (e.g., can be coded single item and 'standardized') |
|  | **Multi Item Perceived Need** | For studies, which measure perceived need: Is this variable measured using a multi-item index? | 0 = No 1 = Yes | E.g., uses more than one question to measure perceived need   Not mutually exclusive (e.g., can be coded multi item and 'standardized') |
|  | **Standardized Perceived Need** | For studies, which measure perceived need: Is this variable measured using a standardized scale or instrument? | 0 = No 1 = Yes | E.g., uses a standardized instrument to measure perceived need (e.g., the PNCQ, Camberwell Needs Assessment, etc.)  Measure should be coded as multi or single item first, then yes or no for standardized. |
|  | **Name of standardized perceived need instrument** | For studies in which the measure of perceived need is standardized, specify the name of the instrument | String variable; write in name of instrument |  |
|  | **Generic or specific service needs** | Is perceived need measured for only one generic service category (e.g., mental health treatment, addiction treatment, healthcare for substance problems) or for one or more specific service categories (e.g., counselling, medication, harm reduction, residential treatment, etc.)? | 0 = Generic 1 = Specific | Examples of generic service categories include: mental health care, mental health treatment, addiction treatment, etc.  Examples of specific service categories include: in-patient treatment, out-patient treatment, counselling, medication, methadone, hospital care, harm reduction services, needle exchange, etc. |
|  | **Help-seeking** | Does the study estimate the proportion of the sample that sought help for problems with substance use or mental health from family or friends? | 0 = No 1 = Yes | Does the study estimate the proportion of the sample that sought help for problems with substance use or mental health from family or friends?  Note 'hypothetical' questions about help seeking *if* help is needed are not coded as informal help seeking. **Not mutually exclusive, e.g., study can estimate more than one perceived need-related variable |
|  | **Single Item Help-Seeking** | For studies, which measure help seeking: Is this variable measured using a single item measure? | 0 = No 1 = Yes | E.g., uses one question to measure help-seeking  Not mutually exclusive (e.g., can be coded single item and 'standardized') |
|  | **Multi Item Help-Seeking** | For studies, which measure help seeking: Is this variable measured using a multi-item index? | 0 = No 1 = Yes | E.g., uses more than one question to measure help-seeking  Not mutually exclusive (e.g., can be coded multi item and 'standardized') |
|  | **Standardized Help-Seeking** | For studies, which measure help seeking: Is this variable measured using a standardized scale or instrument? | 0 = No 1 = Yes | E.g., uses a standardized instrument to measure informal help-seeking   Measure should be coded as multi or single item first, then yes or no for standardized. |
|  | **Specify name of standardized help-seeking instrument** | For studies in which the measure of informal help-seeking is standardized, specify the name of the instrument | String variable; write in name of instrument |  |
|  | **Self-assessed barriers to care** | Does the study report participants’ self-assessed reasons for being unable to access help for substance use and/or mental health problems? | 0 = No 1 = Yes | Does the study report participants’ self-assessed reasons for being unable to access help for substance use and/or mental health problems?  **Not mutually exclusive, e.g., study can estimate more than one perceived need-related variable |
|  | **Single Item Self-assessed Barriers to Care** | For studies, which measure self-assessed barriers to care: Is this variable measured using a single item measure? | 0 = No 1 = Yes | E.g., uses one question to measure self-assessed barriers to care  Not mutually exclusive (e.g., can be coded single item and 'standardized' |
|  | **Multi Item Self-assessed Barriers to Care** | For studies, which measure self-assessed barriers to care: Is this variable measured using a multi-item index? | 0 = No 1 = Yes | E.g., uses more than one question to measure self-assessed barriers to care  Not mutually exclusive (e.g., can be coded multi item and 'standardized') |
|  | **Standardized Self-assessed Barriers to Care** | For studies, which measure self-assessed barriers to care: Is this variable measured using a standardized scale or instrument? | 0 = No 1 = Yes | E.g., uses a standardized instrument to measure self-assessed barriers to care (e.g., the PNCQ),   Measure should be coded as multi or single item first, then yes or no for standardized. |
| **Analysis** | **Specify name of standardized barriers instrument** | For studies in which the measure of self-assessed barriers to care is standardized, specify the name of the instrument | String variable; write in name of instrument |  |
|  | **Hypothesis testing** | Does the perceived need study test one or more explicitly stated hypotheses? | 0 = No 1 = Yes | E.g., The authors state hypotheses tested in their analysis and describe whether their findings are or are not consistent with these hypotheses |
|  | **Generates estimates** | Does the study generate estimates of required services system capacity to meet population need for substance use services? | 0 = No 1 = Yes | Does the study use population survey data on perceived need for care to model the required service system capacity to meet population need for substance use services (e.g., how many treatment spaces would be required, how many physician visits would be required, how many people would need counselling, how many people would need brief interventions, etc.?) |
|  | **General Health and Social Services** | For studies, which generate estimates: are the estimates for the required capacity of one or more general health and social services? | 0 = No 1 = Yes | For studies, which generate estimates: are the estimates for the required capacity of one or more general health and social services?  Examples include: primary care, medication, social services, acute or hospital care, harm reduction, skills training, general counselling, etc.  **Not mutually exclusive, can estimate more than one type of service |
|  | **Specialty addiction and mental health care** | For studies, which generate estimates: are the estimates for the required capacity of one or more specialty health and social services? | 0 = No 1 = Yes | For studies, which generate estimates: are the estimates for the required capacity of one or more specialty health and social services? Examples include: addiction treatment, opioid dependence treatment, psychiatric hospital care, residential substance use treatment programs, etc. **not mutually exclusive, can estimate more than one type of service |
