# Supplementary material for: The role of consumer perspectives in estimating population need for substance use services: a scoping review
Source: BMC Health Serv Res. 2017 Mar 20;17:217. doi: 10.1186/s12913-017-2153-z (PMC5359989; doi:10.1186/s12913-017-2153-z)
Supplement: Additional file 3: — Consumer-defined need studies. All studies assessing consumer perspective on need for substance use services identified through systematic search (n = 217). (DOCX 52 kb) [file 12913_2017_2153_MOESM3_ESM.docx]

Additional File 3: Studies assessing consumer perspective on need for substance use services identified through systematic search (n = 217)

1. Abagiu, A. O., Cavar, Z., Dannon, P., George, P., Habrat, B., Mahomedy, Z., . . . Kastelic, A. (2014). Outcomes from the International Survey Informing Greater Insights in Opioid Dependence Treatment (INSIGHT) project. *Drugs: Education, Prevention & Policy, 21*(6), 440-450. doi:http://dx.doi.org/10.3109/09687637.2014.945511
2. Al-Tayyib, A. A., & Koester, S. (2011). Injection drug users' experience with and attitudes toward methadone clinics in Denver, CO. *Journal of Substance Abuse Treatment, 41*(1), 30-36. doi:<http://dx.doi.org/10.1016/j.jsat.2011.01.009>
3. Alam, F., & Barker, P. (2014). Interruption of medication-assisted treatment for opioid dependence: Insights from the UK. *Drugs and Alcohol Today, 14*(3), 114-125. doi:10.1108/DAT-01-2014-0002
4. Alemagno, S. A. (2001). Women in jail: is substance abuse treatment enough? *American Journal of Public Health, 91*(5), 798-800.
5. Ali, M. M., Teich, J. L., & Mutter, R. (2015). The role of perceived need and health insurance in substance use treatment: implications for the affordable care act. *Journal of Substance Abuse Treatment, 54*, 14-20. doi:<http://dx.doi.org/10.1016/j.jsat.2015.02.002>
6. Andrews, G., Henderson, S., & Hall, W. (2001). Prevalence, comorbidity, disability and service utilisation: Overview of the Australian National Mental Health Survey. *The British Journal of Psychiatry, 178*, 145-153. doi:<http://dx.doi.org/10.1192/bjp.178.2.145>
7. Arun, P., Chavan, B. S., & Kaur, H. (2004). A Study of reasons for not seeking treatment for substance abuse in community. *Indian Journal of Psychiatry, 46*(3), 256-260.
8. Aveyard, H. (1999). Illicit drug use: information-giving strategies requested by students in higher education. *Health Education Journal, 58*(3), 239-248.
9. Baksheev, G. N., Thomas, S. D., & Ogloff, J. R. (2010). Psychiatric disorders and unmet needs in Australian police cells. *Australian & New Zealand Journal of Psychiatry, 44*(11), 1043-1051. doi:<http://dx.doi.org/10.1080/00048674.2010.503650>
10. Baldwin, D. M., Brecht, M. L., Monahan, G., Annon, K., Wellisch, J., & Anglin, M. D. (1995). Perceived need for treatment among pregnant and nonpregnant women arrestees. *Journal of Psychoactive Drugs, 27*(4), 389-399.
11. Barratt, M. J., Cakic, V., & Lenton, S. (2013). Patterns of synthetic cannabinoid use in Australia. *Drug and Alcohol Review, 32*(2), 141-146. doi:<http://dx.doi.org/10.1111/j.1465-3362.2012.00519.x>
12. Becker, W. C., Fiellin, D. A., Merrill, J. O., Schulman, B., Finkelstein, R., Olsen, Y., & Busch, S. H. (2008). Opioid use disorder in the United States: insurance status and treatment access. *Drug & Alcohol Dependence, 94*(1-3), 207-213. doi:<http://dx.doi.org/10.1016/j.drugalcdep.2007.11.018>
13. Belenko, S., Lang, M. A., & O'Connor, L. A. (2003). Self-Reported Psychiatric Treatment Needs Among Felony Drug Offenders. *Journal of Contemporary Criminal Justice, 19*(1), 9-29. doi:<http://dx.doi.org/10.1177/1043986202239740>
14. Bennett, T., & Holloway, K. (2008). Identifying and preventing health problems among young drug-misusing offenders. *Health Education, 108*(3), 247-261. doi:<http://dx.doi.org/10.1108/09654280810867114>
15. Bennett, T., & Wright, R. (1986). Opioid users' attitudes towards and use of NHS clinics, general practitioners and private doctors. *British Journal of Addiction, 81*(6), 757-763.
16. Benyamina, A. (2014). The current status of opioid maintenance treatment in France: a survey of physicians, patients, and out-of-treatment opioid users. *International journal of general medicine, 7*, 449-457. doi:<http://dx.doi.org/10.2147/IJGM.S61014>
17. Blitz, C. L., Wolff, N., & Paap, K. (2006). Availability of behavioral health treatment for women in prison. *Psychiatric Services, 57*(3), 356-360.
18. Bluthenthal, R. N., Kral, A. H., Lorvick, J., & Watters, J. K. (1997). Impact of law enforcement on syringe exchange programs: A look at Oakland and San Francisco. *Medical Anthropology, 18*(1), 61-83. doi:<http://dx.doi.org/10.1080/01459740.1997.9966150>
19. Booth, B. M., Curran, G. M., Han, X., & Edlund, M. J. (2013). Criminal justice and alcohol treatment: results from a national sample. *Journal of Substance Abuse Treatment, 44*(3), 249-255. doi:<http://dx.doi.org/10.1016/j.jsat.2012.07.008>
20. Booth, B. M., Stewart, K. E., Curran, G. M., Cheney, A. M., & Borders, T. F. (2014). Beliefs and attitudes regarding drug treatment: application of the theory of planned behavior in African-American cocaine users. *Addictive Behaviors, 39*(10), 1441-1446. doi:<http://dx.doi.org/10.1016/j.addbeh.2014.05.012>
21. Boothroyd, R. A., Best, K. A., Giard, J. A., Stiles, P. G., Suleski, J., Ort, R., & White, R. (2006). Poor and depressed, the tip of the iceberg: The unmet needs of enrollees in an indigent health care plan. *Administration and Policy in Mental Health and Mental Health Services Research, 33*(2), 172-181. doi:<http://dx.doi.org/10.1007/s10488-006-0030-x>
22. Borders, T. F., Booth, B. M., Stewart, K. E., Cheney, A. M., & Curran, G. M. (2015). Rural/urban residence, access, and perceived need for treatment among African American cocaine users. *Journal of Rural Health, 31*(1), 98-107. doi:<http://dx.doi.org/10.1111/jrh.12092>
23. Bottomley, T., Carnwath, T., Jeacock, J., Wibberley, C., & Smith, M. (1997). Crack cocaine-tailoring services to user need. *Addiction Research, 5*(3), 223-234. doi:<http://dx.doi.org/10.3109/16066359709005263>
24. Brands, B., Leslie, K., Catz-Biro, L., & Li, S. (2005). Heroin use and barriers to treatment in street-involved youth. *Addiction Research & Theory, 13*(5), 477-487. doi:<http://dx.doi.org/10.1080/16066350500150624>
25. Brook, R., Klap, R., Liao, D., & Wells, K. B. (2006). Mental health care for adults with suicide ideation. *General Hospital Psychiatry, 28*(4), 271-277. doi:<http://dx.doi.org/10.1016/j.genhosppsych.2006.01.001>
26. Brubaker, M. D., Amatea, E. A., Torres-Rivera, E., Miller, M., & Nabors, L. (2013). Barriers and supports to substance abuse service use among homeless adults. *Journal of Addictions & Offender Counseling, 34*(2), 81-98.
27. Caldeira, K. M., Kasperski, S. J., Sharma, E., Vincent, K. B., O'Grady, K. E., Wish, E. D., & Arria, A. M. (2009). College students rarely seek help despite serious substance use problems. *Journal of Substance Abuse Treatment, 37*(4), 368-378. doi:<http://dx.doi.org/10.1016/j.jsat.2009.04.005>
28. Carruthers, S., Loxley, W., & Bevan, J. (1998). Changing the habit: Why and how do users change their drug consumption patterns? *Journal of Substance Misuse, 3*(1), 50-54.
29. Chen, L. Y., Crum, R. M., Martins, S. S., Kaufmann, C. N., Strain, E. C., & Mojtabai, R. (2013). Service use and barriers to mental health care among adults with major depression and comorbid substance dependence. *Psychiatric Services, 64*(9), 863-870. doi:<http://dx.doi.org/10.1176/appi.ps.201200289>
30. Chen, L. Y., Strain, E. C., Crum, R. M., & Mojtabai, R. (2013). Gender differences in substance abuse treatment and barriers to care among persons with substance use disorders with and without comorbid major depression. *Journal of Addiction Medicine, 7*(5), 325-334. doi:<http://dx.doi.org/10.1097/ADM.0b013e31829b7afe>
31. Cheng, T. C., & Lo, C. C. (2010). Mental health service and drug treatment utilization: Adolescents with substance use/mental disorders and dual diagnosis. *Journal of Child & Adolescent Substance Abuse, 19*(5), 447-460. doi:<http://dx.doi.org/10.1080/1067828X.2010.515887>

1. Cheng, T. C., & Lo, C. C. (2014). Domestic violence and treatment seeking: a longitudinal study of low-income women and mental health/substance abuse care. *International Journal of Health Services, 44*(4), 735-759.
2. Cheng, T. C., & Robinson, M. A. (2013). Factors leading African Americans and black Caribbeans to use social work services for treating mental and substance use disorders. *Health & Social Work, 38*(2), 99-109.
3. Chitsabesan, P., Rothwell, J., Kenning, C., Law, H., Carter, L. A., Bailey, S., & Clark, A. (2012). Six years on: A prospective cohort study of male juvenile offenders in secure care. *European Child and Adolescent Psychiatry, 21*(6), 339-347. doi:<http://dx.doi.org/10.1007/s00787-012-0266-9>
4. Choi, N. G., DiNitto, D. M., & Marti, C. N. (2014). Treatment use, perceived need, and barriers to seeking treatment for substance abuse and mental health problems among older adults compared to younger adults. *Drug & Alcohol Dependence, 145*, 113-120. doi:<http://dx.doi.org/10.1016/j.drugalcdep.2014.10.004>
5. Choi, N. G., DiNitto, D. M., & Marti, C. N. (2015). Alcohol and other substance use, mental health treatment use, and perceived unmet treatment need: Comparison between baby boomers and older adults. *American Journal on Addictions, 24*(4), 299-307. doi:10.1111/ajad.12225
6. Cohen, E., Feinn, R., Arias, A., & Kranzler, H. R. (2007). Alcohol treatment utilization: Findings from the National Epidemiologic Survey on Alcohol and Related Conditions. *Drug and Alcohol Dependence, 86*(2-3), 214-221. doi:<http://dx.doi.org/10.1016/j.drugalcdep.2006.06.008>
7. Copeland, A. L., Businelle, M. S., Stewart, D. W., Patterson, S. M., Rash, C. J., & Carney, C. E. (2010). Identifying barriers to entering smoking cessation treatment among socioeconomically disadvantaged smokers. *Journal of Smoking Cessation, 5*(2), 164-171. doi:<http://dx.doi.org/10.1375/jsc.5.2.164>
8. Copeland, J., Howard, J., Keogh, T., & Seidler, K. (2003). Patterns and correlates of substance use amongst juvenile detainees in New South Wales 1989-99. *Drug & Alcohol Review, 22*(1), 15-20.
9. Corliss, H. L., Grella, C. E., Mays, V. M., & Cochran, S. D. (2006). Drug Use, Drug Severity, and Help-Seeking Behaviors of Lesbian and Bisexual Women. *Journal of Women's Health, 15*(5), 556-568. doi:<http://dx.doi.org/10.1089/jwh.2006.15.556>
10. Cornelius, J. R., Pringle, J., Jernigan, J., Kirisci, L., & Clark, D. B. (2001). Correlates of mental health service utilization and unmet need among a sample of male adolescents. *Addictive Behaviors, 26*(1), 11-19.
11. Cousineau, M. R. (1997). Health status of and access to health services by residents of urban encampments in Los Angeles. *Journal of Health Care for the Poor & Underserved, 8*(1), 70-82.
12. Cranford, J. A., Eisenberg, D., & Serras, A. M. (2009). Substance use behaviors, mental health problems, and use of mental health services in a probability sample of college students. *Addictive Behaviors, 34*(2), 134-145. doi:<http://dx.doi.org/10.1016/j.addbeh.2008.09.004>
13. Day, M., Devieux, J. G., Reid, S. D., Jones, D. J., Meharris, J., & Malow, R. M. (2004). Risk behaviours and healthcare needs of homeless drug users in Saint Lucia and Trinidad. *ABNF Journal, 15*(6), 121-126.
14. Deering, D. E., Sheridan, J., Sellman, J. D., Adamson, S. J., Pooley, S., Robertson, R., & Henderson, C. (2011). Consumer and treatment provider perspectives on reducing barriers to opioid substitution treatment and improving treatment attractiveness. *Addictive Behaviors, 36*(6), 636-642. doi:<http://dx.doi.org/10.1016/j.addbeh.2011.01.004>
15. Digiusto, E., & Treloar, C. (2007). Equity of access to treatment, and barriers to treatment for illicit drug use in Australia. *Addiction, 102*(6), 958-969.
16. Ding, Y., He, N., Shoptaw, S., Gao, M., & Detels, R. (2014). Severity of club drug dependence and perceived need for treatment among a sample of adult club drug users in Shanghai, China. *Social Psychiatry & Psychiatric Epidemiology, 49*(3), 395-404. doi:<http://dx.doi.org/10.1007/s00127-013-0713-z>
17. Dotinga, A., van den Eijnden, R. J., Bosveld, W., & Garretsen, H. F. (2008). Abstaining, excessive drinking, binge drinking, and help-seeking behavior among Turks in the Netherlands. *Journal of Social Work Practice in the Addictions, 8*(1), 44-64. doi:<http://dx.doi.org/10.1080/15332560802108639>
18. Druss, B. G., Wang, P. S., Sampson, N. A., Olfson, M., Pincus, H. A., Wells, K. B., & Kessler, R. C. (2007). Understanding mental health treatment in persons without mental diagnoses: results from the National Comorbidity Survey Replication. *Archives of General Psychiatry, 64*(10), 1196-1203.
19. Edwards, R., McElduff, P., Jenner, D., Heller, R. F., & Langley, J. (2007). Smoking, smoking cessation, and use of smoking cessation aids and support services in South Derbyshire, England. *Public Health (Elsevier), 121*(5), 321-332. doi:10.1016/j.puhe.2006.11.002
20. Elbogen, E. B., Wagner, H., Johnson, S. C., Kinneer, P., Kang, H., Vasterling, J. J., . . . Beckham, J. C. (2013). Are Iraq and Afghanistan veterans using mental health services? New data from a national random-sample survey. *Psychiatric Services, 64*(2), 134-141. doi:<http://dx.doi.org/10.1176/appi.ps.004792011>
21. Evans, J. D., Wright, D. E., Svanum, S., & Bond, G. R. (2004). Psychiatric disorder and unmet service needs among welfare clients in a representative payee program. *Community Mental Health Journal, 40*(6), 539-548.
22. Fairbairn, N., Milloy, M. J., Zhang, R., Lai, C., Grafstein, E., Kerr, T., & Wood, E. (2012). Emergency department utilization among a cohort of HIV-positive injecting drug users in a Canadian setting. *Journal of Emergency Medicine, 43*(2), 236-243. doi:<http://dx.doi.org/10.1016/j.jemermed.2011.05.020>
23. Falck, R. S., Wang, J., Carlson, R. G., Eddy, M., & Siegal, H. A. (2002). The prevalence and correlates of depressive symptomatology among a community sample of crack-cocaine smokers. *Journal of Psychoactive Drugs, 34*(3), 281-288.
24. Falck, R. S., Wang, J., Carlson, R. G., Krishnan, L. L., Leukefeld, C., & Booth, B. M. (2007). Perceived need for substance abuse treatment among illicit stimulant drug users in rural areas of Ohio, Arkansas, and Kentucky. *Drug & Alcohol Dependence, 91*(2-3), 107-114.
25. Farabee, D., Leukefeld, C. G., & Hays, L. (1998). Accessing drug-abuse treatment: Perceptions of out-of-treatment injectors. *Journal of Drug Issues, 28*(2), 381-394.
26. Ferri, C. P., Gossop, M., Rabe-Hesketh, S., & Laranjeira, R. R. (2002). Differences in factors associated with first treatment entry and treatment re-entry among cocaine users. *Addiction, 97*(7), 825-832. doi:<http://dx.doi.org/10.1046/j.1360-0443.2002.00130.x>
27. Fichter, M. M., Koniarczyk, M., Greifenhagen, A., Koegel, P., Quadflieg, N., Wittchen, H. U., & Wolz, J. (1996). Mental illness in a representative sample of homeless men in Munich, Germany. *European Archives of Psychiatry and Clinical Neuroscience, 246*(4), 185-196. doi:<http://dx.doi.org/10.1007/BF02188952>
28. Fiorentine, R., & Anglin, M. D. (1994). Perceiving need for drug treatment: a look at eight hypotheses. *International Journal of the Addictions, 29*(14), 1835-1854.
29. Fischer, B., Rehm, J., Brissette, S., Brochu, S., Bruneau, J., El-Guebaly, N., . . . Baliunas, D. (2005). Illicit opioid use in Canada: comparing social, health, and drug use characteristics of untreated users in five cities (OPICAN study). *Journal of Urban Health, 82*(2), 250-266.
30. Fountain, J., Howes, S., & Strang, J. (2003). Unmet drug and alcohol service needs of homeless people in London: a complex issue. *Substance Use & Misuse, 38*(3-6), 377-393.
31. Fox, J., Desai, M. M., Britten, K., Lucas, G., Luneau, R., & Rosenthal, M. S. (2012). Mental-health conditions, barriers to care, and productivity loss among officers in an urban police department. *Connecticut Medicine, 76*(9), 525-531.
32. Garrido, M. M., Kane, R. L., Kaas, M., & Kane, R. A. (2009). Perceived need for mental health care among community-dwelling older adults. *Journals of Gerontology Series B-Psychological Sciences & Social Sciences, 64*(6), 704-712. doi:<http://dx.doi.org/10.1093/geronb/gbp073>
33. Garrido, M. M., Kane, R. L., Kaas, M., & Kane, R. A. (2011). Use of mental health care by community-dwelling older adults. *Journal of the American Geriatrics Society, 59*(1), 50-56. doi:<http://dx.doi.org/10.1111/j.1532-5415.2010.03220.x>
34. Gates, P., Copeland, J., Swift, W., & Martin, G. (2012). Barriers and facilitators to cannabis treatment. *Drug & Alcohol Review, 31*(3), 311-319. doi:<http://dx.doi.org/10.1111/j.1465-3362.2011.00313.x>
35. Gil-Rivas, V. (1997). Patterns of drug use and criminal activities among Latino arrestees in California: Treatment and policy implications. *Journal of Psychopathology and Behavioral Assessment, 19*(2), 161-174. doi:<http://dx.doi.org/10.1007/BF02229041>
36. Goldstein, A. L., Henriksen, C. A., Davidov, D. M., Kimber, M., Pitre, N. Y., & Afifi, T. O. (2013). Childhood maltreatment, alcohol use disorders, and treatment utilization in a national sample of emerging adults. *Journal of Studies on Alcohol & Drugs, 74*(2), 185-194.
37. Goldstein, R. B., Olfson, M., Martens, E. G., & Wolk, S. I. (2006). Subjective unmet need for mental health services in depressed children grown up. *Administration & Policy in Mental Health, 33*(6), 666-673.
38. Golub, A., Vazan, P., Bennett, A. S., & Liberty, H. J. (2013). Unmet need for treatment of substance use disorders and serious psychological distress among veterans: a nationwide analysis using the NSDUH. *Military Medicine, 178*(1), 107-114.
39. Grant, B. F. (1997). Barriers to alcoholism treatment: reasons for not seeking treatment in a general population sample. *Journal of Studies on Alcohol, 58*(4), 365-371.
40. Green-Hennessy, S. (2002). Factors associated with receipt of behavioral health services among persons with substance dependence. *Psychiatric Services, 53*(12), 1592-1598. doi:<http://dx.doi.org/10.1176/appi.ps.53.12.1592>
41. Grella, C. E., Karno, M. P., Warda, U. S., Moore, A. A., & Niv, N. (2009). Perceptions of need and help received for substance dependence in a national probability survey. *Psychiatric Services, 60*(8), 1068-1074. doi:<http://dx.doi.org/10.1176/appi.ps.60.8.1068>
42. Guenter, C. D., Fonseca, K., Nielsen, D. M., Wheeler, V. J., & Pim, C. P. (2000). HIV prevalence remains low among Calgary's needle exchange program participants. *Canadian Journal of Public Health. Revue Canadienne de Sante Publique, 91*(2), 129-132.
43. Guerrero, E. G., Villatoro, J. A., Kong, Y., Fleiz, C., Vega, W. A., Strathdee, S. A., & Medina-Mora, M. E. (2014). Barriers to accessing substance abuse treatment in Mexico: national comparative analysis by migration status. *Substance Abuse Treatment, Prevention, & Policy, 9*, 30. doi:<http://dx.doi.org/10.1186/1747-597X-9-30>
44. Hadland, S. E., Kerr, T., Li, K., Montaner, J. S., & Wood, E. (2009). Access to drug and alcohol treatment among a cohort of street-involved youth. *Drug & Alcohol Dependence, 101*(1-2), 1-7. doi:<http://dx.doi.org/10.1016/j.drugalcdep.2008.10.012>
45. Han, B., McKeon, R., & Gfroerer, J. (2014). Suicidal Ideation Among Community-Dwelling Adults in the United States. *American Journal of Public Health, 104*(3), 488-497. doi:10.2105/AJPH.2013.301600
46. Hando, J., Howard, J., & Zibert, E. (1997). Risky drug practices and treatment needs of youth detained in New South Wales juvenile justice centres. *Drug and Alcohol Review, 16*(2), 137-145. doi:<http://dx.doi.org/10.1080/09595239700186431>
47. Hando, J., Topp, L., & Hall, W. (1997). Amphetamine-related harms and treatment preferences of regular amphetamine users in Sydney, Australia. *Drug & Alcohol Dependence, 46*(1-2), 105-113.
48. Harris, K. M., & Edlund, M. J. (2005). Self-medication of mental health problems: new evidence from a national survey. *Health Services Research, 40*(1), 117-134.
49. Hatzenbuehler, M. L., Keyes, K. M., Narrow, W. E., Grant, B. F., & Hasin, D. S. (2008). Racial/ethnic disparities in service utilization for individuals with co-occurring mental health and substance use disorders in the general population: Results from the National Epidemiologic Survey on Alcohol and Related Conditions. *Journal of Clinical Psychiatry, 69*(7), 1112-1121. doi:<http://dx.doi.org/10.4088/JCP.v69n0711>
50. Hedden, S. L., & Gfroerer, J. C. (2011). Correlates of perceiving a need for treatment among adults with substance use disorder: results from a national survey. *Addictive Behaviors, 36*(12), 1213-1222. doi:<http://dx.doi.org/10.1016/j.addbeh.2011.07.026>
51. Iversen, A. C., van Staden, L., Hughes, J. H., Browne, T., Greenberg, N., Hotopf, M., . . . Fear, N. T. (2010). Help-seeking and receipt of treatment among UK service personnel.[Erratum appears in Br J Psychiatry. 2010 Sep;197(3):247-8]. *British Journal of Psychiatry, 197*(2), 149-155. doi:<http://dx.doi.org/10.1192/bjp.bp.109.075762>
52. Kaufmann, C. N., Chen, L.-Y., Crum, R. M., & Mojtabai, R. (2014). Treatment seeking and barriers to treatment for alcohol use in persons with alcohol use disorders and comorbid mood or anxiety disorders. *Social Psychiatry and Psychiatric Epidemiology, 49*(9), 1489-1499. doi:<http://dx.doi.org/10.1007/s00127-013-0740-9>
53. Kelly, B. C., Liu, T., Zhang, G., Hao, W., & Wang, J. (2014). Factors related to psychosocial barriers to drug treatment among Chinese drug users. *Addictive Behaviors, 39*(8), 1265-1271. doi:<http://dx.doi.org/10.1016/j.addbeh.2014.04.012>
54. Kenny, P., Harney, A., Lee, N. K., & Pennay, A. (2011). Treatment utilization and barriers to treatment: Results of a survey of dependent methamphetamine users. *Substance Abuse Treatment, Prevention & Policy, 6*(1), 3-3. doi:10.1186/1747-597X-6-3
55. Kerr, T., Fairbairn, N., Hayashi, K., Suwannawong, P., Kaplan, K., Zhang, R., & Wood, E. (2010). Difficulty accessing syringes and syringe borrowing among injection drug users in Bangkok, Thailand. *Drug & Alcohol Review, 29*(2), 157-161. doi:<http://dx.doi.org/10.1111/j.1465-3362.2009.00093.x>
56. Khampang, R., Assanangkornchai, S., & Teerawattananon, Y. (2015). Perceived barriers to utilise methadone maintenance therapy among male injection drug users in rural areas of southern Thailand. *Drug and Alcohol Review*. doi:10.1111/dar.12268
57. Khan, S., Okuda, M., Hasin, D. S., Secades-Villa, R., Keyes, K., Lin, K. H., . . . Blanco, C. (2013). Gender differences in lifetime alcohol dependence: results from the national epidemiologic survey on alcohol and related conditions. *Alcoholism: Clinical & Experimental Research, 37*(10), 1696-1705. doi:<http://dx.doi.org/10.1111/acer.12158>
58. Kim, J. Y., & Fendrich, M. (2002). Gender differences in juvenile arrestees' drug use, self-reported dependence, and perceived need for treatment. *Psychiatric Services, 53*(1), 70-75.

1. Kissell, A., Taylor, P. J., Walker, J., Lewis, E., Hammond, A., & Amos, T. (2014). Disentangling Alcohol-Related Needs Among Pre-trial Prisoners: A Longitudinal Study. *Alcohol & Alcoholism, 49*(6), 639-644.
2. Kitchin, H. A. (2005). Needing Treatment: A Snapshot of Provincially Incarcerated Adult Offenders in Nova Scotia with a View towards Substance Abuse and Population Health. *Canadian Journal of Criminology and Criminal Justice, 47*(3), 501-525. doi:<http://dx.doi.org/10.3138/cjccj.47.3.501>
3. Ko, J. Y., Martins, S. S., Kuramoto, S. J., & Chilcoat, H. D. (2010). Patterns of alcohol-dependence symptoms using a latent empirical approach: associations with treatment usage and other correlates. *Journal of Studies on Alcohol & Drugs, 71*(6), 870-878.
4. Krausz, R. M., Clarkson, A. F., Strehlau, V., Torchalla, I., Li, K., & Schuetz, C. G. (2013). Mental disorder, service use, and barriers to care among 500 homeless people in 3 different urban settings. *Social Psychiatry & Psychiatric Epidemiology, 48*(8), 1235-1243. doi:<http://dx.doi.org/10.1007/s00127-012-0649-8>
5. Kurtz, S. P., Surratt, H. L., Kiley, M. C., & Inciardi, J. A. (2005). Barriers to health and social services for street-based sex workers. *Journal of Health Care for the Poor & Underserved, 16*(2), 345-361.
6. Kuyper, L. M., Lampinen, T. M., Li, K., Spittal, P. M., Hogg, R. S., Schechter, M. T., & Wood, E. (2004). Factors associated with sex trade involvement among male participants in a prospective study of injection drug users. *Sexually Transmitted Infections, 80*(6), 531-535.
7. Kuyper, L. M., Palepu, A., Kerr, T., Li, K., Miller, C. L., Spittal, P. M., . . . Wood, E. (2005). Factors associated with sex-trade involvement among female injection drug users in a Canadian setting. *Addiction Research & Theory, 13*(2), 193-199. doi:<http://dx.doi.org/10.1080/16066350500053349>
8. Langle, G., Egerter, B., Albrecht, F., Petrasch, M., & Buchkremer, G. (2005). Prevalence of mental illness among homeless men in the community: Approach to a full census in a southern German university town. *Social Psychiatry and Psychiatric Epidemiology, 40*(5), 382-390. doi:<http://dx.doi.org/10.1007/s00127-005-0902-5>
9. Lee, S., Guo, W. J., Tsang, A., He, Y. L., Huang, Y. Q., Liu, Z. R., . . . Kessler, R. C. (2010). Perceived barriers to mental health treatment in metropolitan China. *Psychiatric Services, 61*(12), 1260-1262. doi:<http://dx.doi.org/10.1176/appi.ps.61.12.1260>
10. Lemming, M. R., & Calsyn, R. J. (2006). Ability of the Behavioral Model to Predict Utilization of Five Services by Individuals Suffering from Severe Mental Illness and Homelessness. *Journal of Social Service Research, 32*(3), 153-172. doi:<http://dx.doi.org/10.1300/J079v32n03_09>
11. Lennings, C. J., Kenny, D. T., & Nelson, P. (2006). Substance use and treatment seeking in young offenders on community orders. *Journal of Substance Abuse Treatment, 31*(4), 425-432.
12. Linden, I. A., Torchalla, I., & Krausz, M. (2013). Addiction in maternity: Prevalence of mental illness, substance use, and trauma. *Journal of Aggression, Maltreatment and Trauma, 22*(10), 1070-1084. doi:<http://dx.doi.org/10.1080/10926771.2013.845279>
13. Lipsky, S., Caetano, R., & Roy-Byrne, P. (2011). Triple jeopardy: impact of partner violence perpetration, mental health and substance use on perceived unmet need for mental health care among men. *Social Psychiatry & Psychiatric Epidemiology, 46*(9), 843-852. doi:<http://dx.doi.org/10.1007/s00127-010-0258-3>
14. Lo, C. C. (2004). Sociodemographic factors, drug abuse, and other crimes: How they vary among male and female arrestees. *Journal of Criminal Justice, 32*(5), 399-409. doi:<http://dx.doi.org/10.1016/j.jcrimjus.2004.06.002>
15. Lo, C. C., & Cheng, T. C. (2013). American Youths' Access to Substance Abuse Treatment: Does Type of Treatment Facility Matter? *Journal of Child & Adolescent Substance Abuse, 22*(3), 191-213. doi:10.1080/1067828X.2012.733582
16. Lo, C. C., & Stephens, R. C. (2000). Drugs and prisoners: treatment needs on entering prison. *American Journal of Drug & Alcohol Abuse, 26*(2), 229-245.
17. Lo, C. C., & Stephens, R. C. (2002). Arrestees' perceived needs for substance-specific treatment: exploring urban-rural differences. *American Journal of Drug & Alcohol Abuse, 28*(4), 623-642.
18. Lofwall, M. R., & Havens, J. R. (2012). Inability to access buprenorphine treatment as a risk factor for using diverted buprenorphine. *Drug & Alcohol Dependence, 126*(3), 379-383. doi:<http://dx.doi.org/10.1016/j.drugalcdep.2012.05.025>
19. Longshore, D. (1998). Desire for help among drug-using Mexican-American arrestees. *Substance Use & Misuse, 33*(6), 1387-1406. doi:<http://dx.doi.org/10.3109/10826089809062223>
20. Longshore, D., Grills, C., Anglin, M., & Annon, K. (1997). Desire for help among African-American drug users. *Journal of Drug Issues, 27*(4), 755-770.
21. Longshore, D., Hsieh, S. C., Anglin, M. D., & Annon, T. A. (1992). Ethnic patterns in drug abuse treatment utilization. *Journal of Mental Health Administration, 19*(3), 268-277.
22. Lopez, V. A. (2003). Perceived need for substance abuse treatment among White, Hispanic, and Black juvenile arrestees. *Journal of Ethnicity in Substance Abuse, 2*(4), 1-17. doi:<http://dx.doi.org/10.1300/J233v02n04_01>
23. MacMaster, S. A. (2013). Perceptions of need, service use, and barriers to service access among female methamphetamine users in rural Appalachia. *Social Work in Public Health, 28*(2), 109-118. doi:<http://dx.doi.org/10.1080/19371918.2011.560820>
24. Maher, L., Sargent, P., Higgs, P., Crofts, N., Kelsall, J., & Le, T. T. (2001). Risk behaviours of young Indo-Chinese injecting drug users in Sydney and Melbourne. *Australian & New Zealand Journal of Public Health, 25*(1), 50-54.
25. Mann, A., Spjeldnes, S., & Yamatani, H. (2013). Male County Jail Inmates: A Profile and Self-Reported Human Service Needs by Race. *Journal of Evidence-Based Social Work, 10*(4), 265-275. doi:10.1080/15433714.2011.561128
26. Mason, D., Birmingham, L., & Grubin, D. (1997). Substance use in remand prisoners: a consecutive case study. *BMJ, 315*(7099), 18-21.
27. Mason, M. J., Keyser-Marcus, L., Snipes, D., Benotsch, E., & Sood, B. (2013). Perceived mental health treatment need and substance use correlates among young adults. *Psychiatric Services, 64*(9), 871-877. doi:<http://dx.doi.org/10.1176/appi.ps.201200159>
28. McAuliffe, W. E., Breer, P., Ahmadifar, N. W., & Spino, C. (1991). Assessment of drug abuser treatment needs in Rhode Island. *American Journal of Public Health, 81*(3), 365-371.
29. McCoy, C. B., Metsch, L. R., Chitwood, D. D., & Miles, C. (2001). Drug use and barriers to use of health care services. *Substance Use & Misuse, 36*(6-7), 789-806.
30. Meadows, G. N., & Burgess, P. M. (2009). Perceived need for mental health care: findings from the 2007 Australian Survey of Mental Health and Wellbeing. *Australian & New Zealand Journal of Psychiatry, 43*(7), 624-634. doi:<http://dx.doi.org/10.1080/00048670902970866>
31. Melchior, M., Prokofyeva, E., Younes, N., Surkan, P. J., & Martins, S. S. (2014). Treatment for illegal drug use disorders: the role of comorbid mood and anxiety disorders. *BMC Psychiatry, 14*, 89. doi:<http://dx.doi.org/10.1186/1471-244X-14-89>
32. Mojtabai, R. (2005). Use of specialty substance abuse and mental health services in adults with substance use disorders in the community. *Drug and Alcohol Dependence, 78*(3), 345-354. doi:<http://dx.doi.org/10.1016/j.drugalcdep.2004.12.003>
33. Mojtabai, R., & Crum, R. M. (2013). Perceived unmet need for alcohol and drug use treatments and future use of services: results from a longitudinal study. *Drug & Alcohol Dependence, 127*(1-3), 59-64. doi:<http://dx.doi.org/10.1016/j.drugalcdep.2012.06.012>
34. Mojtabai, R., Olfson, M., & Mechanic, D. (2002). Perceived need and help-seeking in adults with mood, anxiety, or substance use disorder. *Archives of General Psychiatry, 59*(1), 77-84. doi:<http://dx.doi.org/10.1001/archpsyc.59.1.77>
35. Mojtabai, R., Olfson, M., Sampson, N., Jin, R., Druss, B., Wang, P., . . . Kessler, R. (2011). Barriers to mental health treatment: Results from the National Comorbidity Survey Replication. *Psychological Medicine, 41*(8), 1751-1761. doi:<http://dx.doi.org/10.1017/S0033291710002291>
36. Mowbray, O., Perron, B. E., Bohnert, A. S., Krentzman, A. R., & Vaughn, M. G. (2010). Service use and barriers to care among heroin users: results from a national survey. *American Journal of Drug & Alcohol Abuse, 36*(6), 305-310. doi:<http://dx.doi.org/10.3109/00952990.2010.503824>
37. Mullings, J. L., Hartley, D. J., & Marquart, J. W. (2004). Exploring the relationship between alcohol use, childhood maltreatment, and treatment needs among female prisoners. *Substance Use & Misuse, 39*(2), 277-305.
38. Mulvaney-Day, N., DeAngelo, D., Chen, C. N., Cook, B. L., & Alegria, M. (2012). Unmet need for treatment for substance use disorders across race and ethnicity. *Drug & Alcohol Dependence, 125 Suppl 1*, S44-50. doi:<http://dx.doi.org/10.1016/j.drugalcdep.2012.05.005>
39. Myers, B. (2013). Barriers to alcohol and other drug treatment use among Black African and Coloured South Africans. *BMC Health Services Research, 13*, 177. doi:<http://dx.doi.org/10.1186/1472-6963-13-177>
40. Myers, B., Kline, T. L., Doherty, I. A., Carney, T., & Wechsberg, W. M. (2014). Perceived need for substance use treatment among young women from disadvantaged communities in Cape Town, South Africa. *BMC Psychiatry, 14*, 100. doi:<http://dx.doi.org/10.1186/1471-244X-14-100>
41. Myers, B., Louw, J., & Pasche, S. (2011). Gender differences in barriers to alcohol and other drug treatment in Cape Town, South Africa. *African Journal of Psychiatry, 14*(2), 146-153.
42. Myers, B. J., Louw, J., & Pasche, S. C. (2010). Inequitable access to substance abuse treatment services in Cape Town, South Africa. *Substance Abuse Treatment, Prevention, & Policy, 5*, 28. doi:<http://dx.doi.org/10.1186/1747-597X-5-28>
43. Narevic, E., Garrity, T. F., Schoenberg, N. E., Hiller, M. L., Webster, J. M., Leukefeld, C. G., & Staton Tindall, M. (2006). Factors predicting unmet health services needs among incarcerated substance users. *Substance Use & Misuse, 41*(8), 1077-1094.
44. Ndetei, D. M., Khasakhala, L., Ong'echa, F. A., Kokonya, D., Mutiso, V., Kuria, M., . . . Akanga, S. (2008). A study of drug use in five urban centres in Kenya. *African Journal of Drug and Alcohol Studies, 7*(1), 17-26.
45. North, C. S., & Smith, E. M. (1993). A systematic study of mental health services utilization by homeless men and women. *Social Psychiatry and Psychiatric Epidemiology, 28*(2), 77-83. doi:<http://dx.doi.org/10.1007/BF00802096>
46. Novak, S. P., & Kral, A. H. (2011). Comparing injection and non-injection routes of administration for heroin, methamphetamine, and cocaine users in the United States. *Journal of Addictive Diseases, 30*(3), 248-257. doi:<http://dx.doi.org/10.1080/10550887.2011.581989>
47. O'Toole, T. P., Conde-Martel, A., Gibbon, J. L., Hanusa, B. H., Freyder, P. J., & Fine, M. J. (2007). Where do people go when they first become homeless? A survey of homeless adults in the USA. *Health & Social Care in the Community, 15*(5), 446-453. doi:<http://dx.doi.org/10.1111/j.1365-2524.2007.00703.x>
48. O'Toole, T. P., Freyder, P. J., Gibbon, J. L., Hanusa, B. J., Seltzer, D., & Fine, M. J. (2004). ASAM Patient Placement Criteria treatment levels: do they correspond to care actually received by homeless substance abusing adults? *Journal of Addictive Diseases, 23*(1), 1-15.
49. Ogborne, A. C., & DeWitt, D. J. (1999). Lifetime use of professional and community services for help with drinking: Results from a Canadian population survey. *Journal of Studies on Alcohol, 60*(6), 867-872.
50. Ojeda, V. D., & McGuire, T. G. (2006). Gender and racial/ethnic differences in use of outpatient mental health and substance use services by depressed adults. *Psychiatric Quarterly, 77*(3), 211-222.
51. Oleski, J., Mota, N., Cox, B. J., & Sareen, J. (2010). Perceived need for care, help seeking, and perceived barriers to care for alcohol use disorders in a national sample. *Psychiatric Services, 61*(12), 1223-1231. doi:<http://dx.doi.org/10.1176/appi.ps.61.12.1223>
52. Padgett, D., Struening, E. L., & Andrews, H. (1990). Factors affecting the use of medical, mental health, alcohol, and drug treatment services by homeless adults. *Medical Care, 28*(9), 805-821.
53. Palepu, A., Gadermann, A., Hubley, A. M., Farrell, S., Gogosis, E., Aubry, T., & Hwang, S. W. (2013). Substance use and access to health care and addiction treatment among homeless and vulnerably housed persons in three Canadian cities. *PLoS ONE [Electronic Resource], 8*(10), e75133. doi:<http://dx.doi.org/10.1371/journal.pone.0075133>
54. Park, S., Cho, M. J., Hong, J. P., Sohn, J. H., Lee, H. W., & Park, J. I. (2012). Comparison of treated and untreated alcohol dependence in a nationwide sample of Korean adults. *Addiction Research & Theory, 20*(2), 125-132. doi:10.3109/16066359.2011.580066
55. Perron, B. E., Mowbray, O., Bier, S., Vaughn, M. G., Krentzman, A., & Howard, M. O. (2011). Service use and treatment barriers among inhalant users. *Journal of Psychoactive Drugs, 43*(1), 69-75. doi:<http://dx.doi.org/10.1080/02791072.2011.566504>
56. Perron, B. E., Mowbray, O. P., Glass, J. E., Delva, J., Vaughn, M. G., & Howard, M. O. (2009). Differences in service utilization and barriers among Blacks, Hispanics, and Whites with drug use disorders. *Substance Abuse Treatment, Prevention, & Policy, 4*, 3. doi:<http://dx.doi.org/10.1186/1747-597X-4-3>
57. Phillips, M., DeBeck, K., Desjarlais, T., Morrison, T., Feng, C., Kerr, T., & Wood, E. (2014). Inability to access addiction treatment among street-involved youth in a Canadian setting. *Substance Use & Misuse, 49*(10), 1233-1240. doi:<http://dx.doi.org/10.3109/10826084.2014.891618>
58. Pollini, R. A., McCall, L., Mehta, S. H., Vlahov, D., & Strathdee, S. A. (2006). Non-fatal overdose and subsequent drug treatment among injection drug users. *Drug & Alcohol Dependence, 83*(2), 104-110.
59. Power, R., Hartnoll, R., & Chalmers, C. (1992). Help-seeking among illicit drug users: Some differences between a treatment and nontreatment sample. *International Journal of the Addictions, 27*(8), 887-904.
60. Pridemore, W. A., Damphousse, K. R., & Moore, R. K. (2007). Interview mode effects on estimates of need for alcohol and drug treatment among welfare recipients: Evidence from a quasi-experiment. *Journal of Experimental Criminology, 3*(4), 323-336. doi:<http://dx.doi.org/10.1007/s11292-007-9041-1>
61. Quinn, B., Stoove, M., & Dietze, P. (2013). Factors associated with professional support access among a prospective cohort of methamphetamine users. *Journal of Substance Abuse Treatment, 45*(2), 235-241. doi:<http://dx.doi.org/10.1016/j.jsat.2013.02.003>
62. Quinn, B., Stoove, M., Papanastasiou, C., & Dietze, P. (2013). An exploration of self-perceived non-problematic use as a barrier to professional support for methamphetamine users. *International Journal of Drug Policy, 24*(6), 619-623. doi:<http://dx.doi.org/10.1016/j.drugpo.2013.05.015>
63. Reavley, N. J., Yap, M. B., Wright, A., & Jorm, A. F. (2011). Actions taken by young people to deal with mental disorders: Findings from an Australian national survey of youth. *Early Intervention in Psychiatry, 5*(4), 335-342. doi:<http://dx.doi.org/10.1111/j.1751-7893.2011.00292.x>
64. Reddon, H., Wood, E., Tyndall, M., Lai, C., Hogg, R., Montaner, J., & Kerr, T. (2011). Use of North America's First Medically Supervised Safer Injecting Facility Among HIV-Positive Injection Drug Users. *AIDS Education & Prevention, 23*(5), 412-422. doi:10.1521/aeap.2011.23.5.412
65. Regier, D. A., Narrow, W. E., Rae, D. S., Manderscheid, R. W., Locke, B. Z., & Goodwin, F. K. (1993). The de facto US mental and addictive disorders service system: Epidemiologic Catchment Area prospective 1-year prevalence rates of disorders and services. *Archives of General Psychiatry, 50*(2), 85-94. doi:<http://dx.doi.org/10.1001/archpsyc.1993.01820140007001>
66. Robertson, M. J., Zlotnick, C., & Westerfelt, A. (1997). Drug use disorders and treatment contact among homeless adults in Alameda County, California. *American Journal of Public Health, 87*(2), 221-228.
67. Rosen, D., Tolman, R. M., & Warner, L. A. (2004). Low-income women's use of substance abuse and mental health services. *Journal of Health Care for the Poor & Underserved, 15*(2), 206-219.
68. Rosenthal, D., Mallett, S., Milburn, N., & Rotheram-Borus, M. J. (2008). Drug use among homeless young people in Los Angeles and Melbourne. *Journal of Adolescent Health, 43*(3), 296-305. doi:<http://dx.doi.org/10.1016/j.jadohealth.2008.06.002>
69. Sakai, J. T., Ho, P. M., Shore, J. H., Risk, N. K., & Price, R. K. (2005). Asians in the United States: substance dependence and use of substance-dependence treatment. *Journal of Substance Abuse Treatment, 29*(2), 75-84. doi:10.1016/j.jsat.2005.04.002
70. Santos Cruz, M., Andrade, T., Bastos, F. I., Leal, E., Bertoni, N., Lipman, L., . . . Fischer, B. (2013). Patterns, determinants and barriers of health and social service utilization among young urban crack users in Brazil. *BMC Health Services Research, 13*, 536. doi:<http://dx.doi.org/10.1186/1472-6963-13-536>
71. Santos Cruz, M., Andrade, T., Bastos, F. I., Leal, E., Bertoni, N., Villar, L. M., . . . Fischer, B. (2013). Key drug use, health and socio-economic characteristics of young crack users in two Brazilian cities. *International Journal of Drug Policy, 24*(5), 432-438. doi:<http://dx.doi.org/10.1016/j.drugpo.2013.03.012>
72. Sareen, J., Belik, S. L., Stein, M. B., & Asmundson, G. J. G. (2010). Correlates of perceived need for mental health care among active military personnel. *Psychiatric Services, 61*(1), 50-57. doi:<http://dx.doi.org/10.1176/appi.ps.61.1.50>
73. Sareen, J., Cox, B. J., Afifi, T. O., Stein, M. B., Belik, S. L., Meadows, G., & Asmundson, G. J. G. (2007). Combat and peacekeeping operations in relation to prevalence of mental disorders and perceived need for mental health care: Findings from a large representative sample of military personnel. *Archives of General Psychiatry, 64*(7), 843-852. doi:<http://dx.doi.org/10.1001/archpsyc.64.7.843>
74. Sareen, J., Cox, B. J., Afifi, T. O., Yu, B. N., & Stein, M. B. (2005). Mental health service use in a nationally representative Canadian survey. *Canadian Journal of Psychiatry, 50*(12), 753-761.
75. Sareen, J., Stein, M. B., Campbell, D. W., Hassard, T., & Menec, V. (2005). The relation between perceived need for mental health treatment, DSM diagnosis, and quality of life: a Canadian population-based survey. *Canadian Journal of Psychiatry - Revue Canadienne de Psychiatrie, 50*(2), 87-94.
76. Saunders, S. M., Zygowicz, K. M., & D'Angelo, B. R. (2006). Person-related and treatment-related barriers to alcohol treatment. *Journal of Substance Abuse Treatment, 30*(3), 261-270.
77. Schmidt, L. A., Ye, Y., Greenfield, T. K., & Bond, J. (2007). Ethnic disparities in clinical severity and services for alcohol problems: results from the National Alcohol Survey. *Alcoholism: Clinical & Experimental Research, 31*(1), 48-56.
78. Sevigny, E. L., & Coontz, P. D. (2008). Patterns of substance involvement and criminal behavior: a gender-based cluster analysis of Pennsylvania arrestees. *International Journal of Offender Therapy & Comparative Criminology, 52*(4), 435-453.
79. Shannon, K., Rusch, M., Shoveller, J., Alexson, D., Gibson, K., Tyndall, M. W., & Maka Project, P. (2008). Mapping violence and policing as an environmental-structural barrier to health service and syringe availability among substance-using women in street-level sex work. *International Journal of Drug Policy, 19*(2), 140-147. doi:<http://dx.doi.org/10.1016/j.drugpo.2007.11.024>
80. Siebert, D. C. (2005). Help seeking for AOD misuse among social workers: patterns, barriers, and implications. *Social Work, 50*(1), 65-75.
81. Siegal, H. A., Draus, P. J., Carlson, R. G., Falck, R. S., & Wang, J. (2006). Perspectives on health among adult users of illicit stimulant drugs in rural Ohio. *Journal of Rural Health, 22*(2), 169-173.
82. Siegal, H. A., Falck, R. S., Wang, J., & Carlson, R. G. (2002). Predictors of drug abuse treatment entry among crack-cocaine smokers. *Drug & Alcohol Dependence, 68*(2), 159-166.
83. Spillane, N. S., Greenfield, B., Venner, K., & Kahler, C. W. (2015). Alcohol use among reserve-dwelling adult First Nation members: Use, problems, and intention to change drinking behavior. *Addictive Behaviors, 41*, 232-237. doi:10.1016/j.addbeh.2014.10.015
84. Stajduhar, K. I., Poffenroth, L., Wong, E., Archibald, C. P., Sutherland, D., & Rekart, M. (2004). Missed opportunities: Injection drug use and HIV/AIDS in Victoria, Canada. *International Journal of Drug Policy, 15*(3), 171-181. doi:<http://dx.doi.org/10.1016/j.drugpo.2004.01.001>
85. Stewart, D. (2009). Drug use and perceived treatment need among newly sentenced prisoners in England and Wales. *Addiction, 104*(2), 243-247. doi:<http://dx.doi.org/10.1111/j.1360-0443.2008.02439.x>
86. Stockdale, S. E., Klap, R., Belin, T. R., Zhang, L., & Wells, K. B. (2006). Longitudinal patterns of alcohol, drug, and mental health need and care in a national sample of U.S. adults. *Psychiatric Services, 57*(1), 93-99.
87. Stockdale, S. E., Tang, L., Zhang, L., Belin, T. R., & Wells, K. B. (2007). The effects of health sector market factors and vulnerable group membership on access to alcohol, drug, and mental health care. *Health Services Research, 42*(3 Pt 1), 1020-1041.
88. Struening, E., Padgett, D. K., Pittman, J., Cordova, P., & Jones, M. (1991). A typology based on measures of substance abuse and mental disorder. *Journal of Addictive Diseases, 11*(1), 99-117.
89. Sturm, R., & Sherbourne, C. D. (2001). Are barriers to mental health and substance abuse care still rising? *Journal of Behavioral Health Services & Research, 28*(1), 81-88.
90. Swartz, J. A., & Lurigio, A. J. (2005). Detecting serious mental illness among substance abusers: use of the K6 screening scale. *Journal of Evidence-Based Social Work, 2*(1-2), 113-135.
91. Takeuchi, D. T., Leaf, P. J., & Kuo, H. S. (1988). Ethnic differences in the perception of barriers to help-seeking. *Social Psychiatry & Psychiatric Epidemiology, 23*(4), 273-280.
92. Ti, L., Buxton, J., Wood, E., Shannon, K., Zhang, R., Montaner, J., & Kerr, T. (2012). Factors associated with difficulty accessing crack cocaine pipes in a Canadian setting. *Drug & Alcohol Review, 31*(7), 890-896. doi:<http://dx.doi.org/10.1111/j.1465-3362.2012.00446.x>
93. Ti, L., Buxton, J., Wood, E., Zhang, R., Montaner, J., & Kerr, T. (2011). Difficulty accessing crack pipes and crack pipe sharing among people who use drugs in Vancouver, Canada. *Substance Abuse Treatment, Prevention, & Policy, 6*, 34. doi:<http://dx.doi.org/10.1186/1747-597X-6-34>
94. Todd, C. S., Abed, A. M., Scott, P. T., Botros, B. A., Safi, N., Earhart, K. C., & Strathdee, S. A. (2008). Correlates of receptive and distributive needle sharing among injection drug users in Kabul, Afghanistan. *American Journal of Drug & Alcohol Abuse, 34*(1), 91-100.
95. Treloar, C., & Cao, W. (2005). Barriers to use of Needle and Syringe Programmes in a high drug use area of Sydney, New South Wales. *International Journal of Drug Policy, 16*(5), 308-315. doi:<http://dx.doi.org/10.1016/j.drugpo.2005.06.005>
96. Tucker, J. A. (1995). Predictors of help-seeking and the temporal relationship of help to recovery among treated and untreated recovered problem drinkers. *Addiction, 90*(6), 805-809. doi:<http://dx.doi.org/10.1111/j.1360-0443.1995.tb02228.x>
97. Tucker, J. S., Wenzel, S. L., Golinelli, D., Zhou, A., & Green, H. D., Jr. (2011). Predictors of substance abuse treatment need and receipt among homeless women. *Journal of Substance Abuse Treatment, 40*(3), 287-294. doi:<http://dx.doi.org/10.1016/j.jsat.2010.11.006>
98. Ullman, S. E., & Najdowski, C. J. (2010). Alcohol-related help-seeking in problem drinking women sexual assault survivors. *Substance Use & Misuse, 45*(3), 341-353. doi:<http://dx.doi.org/10.3109/10826080903443644>
99. Urbanoski, K. A., Cairney, J., Bassani, D. G., & Rush, B. R. (2008). Perceived unmet need for mental health care for Canadians with co-occurring mental and substance use disorders. *Psychiatric Services, 59*(3), 283-289. doi:<http://dx.doi.org/10.1176/appi.ps.59.3.283>
100. Urbanoski, K. A., Rush, B. R., Wild, T. C., Bassani, D. G., & Castel, S. (2007). Use of mental health care services by Canadians with co-occurring substance dependence and mental disorders. *Psychiatric Services, 58*(7), 962-969.
101. van der Pol, P., Liebregts, N., de Graaf, R., Korf, D. J., van den Brink, W., & van Laar, M. (2013). Facilitators and barriers in treatment seeking for cannabis dependence. *Drug & Alcohol Dependence, 133*(2), 776-780. doi:<http://dx.doi.org/10.1016/j.drugalcdep.2013.08.011>
102. Venner, K. L., Greenfield, B. L., Vicuna, B., Munoz, R., Bhatt, S., & O'Keefe, V. (2012). "I'm not one of them": barriers to help-seeking among American Indians with alcohol dependence. *Cultural Diversity & Ethnic Minority Psychology, 18*(4), 352-362. doi:<http://dx.doi.org/10.1037/a0029757>
103. Villatoro, A. P., Morales, E. S., & Mays, V. M. (2014). Family culture in mental health help-seeking and utilization in a nationally representative sample of Latinos in the United States: The NLAAS. *American Journal of Orthopsychiatry, 84*(4), 353-363. doi:<http://dx.doi.org/10.1037/h0099844>
104. Vincent, N., Shoobridge, J., Ask, A., Allsop, S., & Ali, R. (1999). Characteristics of amphetamine users seeking information, help and treatment in Adelaide, South Australia. *Drug and Alcohol Review, 18*(1), 63-73. doi:<http://dx.doi.org/10.1080/09595239996770>
105. Wallace, C., Galloway, T., McKetin, R., Kelly, E., & Leary, J. (2009). Methamphetamine use, dependence and treatment access in rural and regional North Coast of New South Wales, Australia. *Drug & Alcohol Review, 28*(6), 592-599. doi:<http://dx.doi.org/10.1111/j.1465-3362.2008.00016.x>
106. Wang, J. (2006). Perceived Barriers To Mental Health Service Use Among Individuals With Mental Disorders in the Canadian General Population. *Medical Care, 44*(2), 192-195. doi:<http://dx.doi.org/10.1097/01.mlr.0000196954.67658.95>
107. Watkins, K. E., Burnam, A., Kung, F. Y., & Paddock, S. (2001). A national survey of care for persons with co-occurring mental and substance use disorders. *Psychiatric Services, 52*(8), 1062-1068.
108. Weber, D., Wolff, L. S., Orleans, T., Mockenhaupt, R. E., Massett, H. A., & Vose, K. K. (2007). Smokers' attitude and behaviors related to consumer demand for cessation counseling in the medical care setting. *Nicotine & Tobacco Research, 9*(5), 571-580. doi:<http://dx.doi.org/10.1080/14622200701189024>
109. Wechsberg, W. M., Wu, L. T., Zule, W. A., Parry, C. D., Browne, F. A., Luseno, W. K., . . . Gentry, A. (2009). Substance abuse, treatment needs and access among female sex workers and non-sex workers in Pretoria, South Africa. *Substance Abuse Treatment, Prevention, & Policy, 4*, 11. doi:<http://dx.doi.org/10.1186/1747-597X-4-11>
110. Weissman, G., Melchior, L., Huba, G., Smereck, G., Needle, R., McCarthy, S., . . . et al. (1995). Women living with drug abuse and HIV disease: drug abuse treatment access and secondary prevention issues. *Journal of Psychoactive Drugs, 27*(4), 401-411.
111. Wells, J., Horwood, L., & Fergusson, D. M. (2007). Reasons why young adults do or do not seek help for alcohol problems. *Australian and New Zealand Journal of Psychiatry, 41*(12), 1005-1012. doi:<http://dx.doi.org/10.1080/00048670701691218>
112. Wells, K., Klap, R., Koike, A., & Sherbourne, C. (2001). Ethnic disparities in unmet need for alcoholism, drug abuse, and mental health care. *American Journal of Psychiatry, 158*(12), 2027-2032.
113. Wells, K. B., Sherbourne, C. D., Sturm, R., Young, A. S., & Burnam, M. A. (2002). Alcohol, drug abuse, and mental health care for uninsured and insured adults. *Health Services Research, 37*(4), 1055-1066.
114. Wenzel, S. L., Audrey Burnam, M., Koegel, P., Morton, S. C., Miu, A., Jinnett, K. J., & Greer Sullivan, J. (2001). Access to inpatient or residential substance abuse treatment among homeless adults with alcohol or other drug use disorders. *Medical Care, 39*(11), 1158-1169.
115. Whealin, J. M., Stotzer, R. L., Pietrzak, R. H., Vogt, D., Shore, J., Morland, L., & Southwick, S. M. (2014). Deployment-related sequelae and treatment utilization in rural and urban war veterans in Hawaii. *Psychological Services, 11*(1), 114-123. doi:<http://dx.doi.org/10.1037/a0032782>
116. Wong, E. C., Marshall, G. N., Schell, T. L., Elliott, M. N., Hambarsoomians, K., Chun, C. A., & Berthold, S. M. (2006). Barriers to mental health care utilization for U.S. Cambodian refugees. *Journal of Consulting and Clinical Psychology, 74*(6), 1116-1120. doi:<http://dx.doi.org/10.1037/0022-006X.74.6.1116>
117. Wood, E., Li, K., Palepu, A., Marsh, D. C., Schechter, M. T., Hogg, R. S., . . . Kerr, T. (2005). Sociodemographic disparities in access to addiction treatment among a cohort of Vancouver injection drug users. *Substance Use & Misuse, 40*(8), 1153-1167.
118. Wood, E., Spittal, P., Li, K., Kerr, T., Miller, C. L., Hogg, R. S., . . . Schechter, M. T. (2004). Inability to access addiction treatment and risk of HIV infection among injection drug users. *Journal of Acquired Immune Deficiency Syndromes: JAIDS, 36*(2), 750-754.
119. Wood, E., Tyndall, M. W., Spittal, P. M., Li, K., Hogg, R. S., O'Shaughnessy, M. V., & Schechter, M. T. (2002). Needle exchange and difficulty with needle access during an ongoing HIV epidemic. *International Journal of Drug Policy, 13*(2), 95-102. doi:<http://dx.doi.org/10.1016/S0955-3959%2802%2900008-7>
120. Woodward, A. T., Taylor, R. J., Bullard, K. M., Neighbors, H. W., Chatters, L. M., & Jackson, J. S. (2008). Use of professional and informal support by African Americans and Caribbean blacks with mental disorders. *Psychiatric Services, 59*(11), 1292-1298. doi:<http://dx.doi.org/10.1176/appi.ps.59.11.1292>
121. Woodward, A. T., Taylor, R. J., & Chatters, L. M. (2011). Use of professional and informal support by Black men with mental disorders. *Research on Social Work Practice, 21*(3), 328-336. doi:<http://dx.doi.org/10.1177/1049731510388668>
122. Wu, E., El-Bassel, N., Gilbert, L., Hess, L., Lee, H. N., & Rowell, T. L. (2012). Prior incarceration and barriers to receipt of services among entrants to alternative to incarceration programs: a gender-based disparity. *Journal of Urban Health, 89*(2), 384-395. doi:<http://dx.doi.org/10.1007/s11524-011-9665-3>
123. Wu, L. T., Blazer, D. G., Li, T. K., & Woody, G. E. (2011). Treatment use and barriers among adolescents with prescription opioid use disorders. *Addictive Behaviors, 36*(12), 1233-1239. doi:<http://dx.doi.org/10.1016/j.addbeh.2011.07.033>
124. Wu, L. T., Pilowsky, D. J., Schlenger, W. E., & Hasin, D. (2007). Alcohol use disorders and the use of treatment services among college-age young adults. *Psychiatric Services, 58*(2), 192-200.
125. Wu, L. T., & Ringwalt, C. L. (2004). Alcohol dependence and use of treatment services among women in the community. *American Journal of Psychiatry, 161*(10), 1790-1797.
126. Wu, L. T., & Ringwalt, C. L. (2006). Use of alcohol treatment and mental health services among adolescents with alcohol use disorders. *Psychiatric Services, 57*(1), 84-92.
127. Yeung, P., & Greenwald, S. (1992). Jewish Americans and mental health: Results of the NIMH Epidemiologic Catchment Area Study. *Social Psychiatry and Psychiatric Epidemiology, 27*(6), 292-297.
128. Zemore, S. E., Mulia, N., Yu, Y., Borges, G., & Greenfield, T. K. (2009). Gender, acculturation, and other barriers to alcohol treatment utilization among Latinos in three National Alcohol Surveys. *Journal of Substance Abuse Treatment, 36*(4), 446-456. doi:<http://dx.doi.org/10.1016/j.jsat.2008.09.005>
